# Analysis of Multi-Zone Reaction Mechanisms in BOF Steelmaking and Comprehensive Simulation

**DOI:** 10.3390/ma18051038

**Published:** 2025-02-26

**Authors:** Zicheng Xin, Qing Liu, Jiangshan Zhang, Wenhui Lin

**Affiliations:** 1 State Key Laboratory of Advanced Metallurgy, University of Science and Technology Beijing, Beijing 100083, China; sklxzc@163.com (Z.X.); zjsustb@163.com (J.Z.); 2 School of Automation and Electrical Engineering, University of Science and Technology Beijing, Beijing 100083, China; 3 Jiangsu Jinheng Information Technology Co., Ltd., Nanjing 210031, China

**Keywords:** BOF steelmaking, multi-zone reaction, mechanism analysis, comprehensive simulation

## Abstract

The BOF steelmaking process involves complex physical and chemical reactions, making precise control challenging when relying solely on human experience. Therefore, understanding the reaction mechanisms and developing simulation models for the BOF process are crucial for enhancing control accuracy and advancing intelligent steelmaking. In this study, the physical and chemical behaviors in various reaction zones were first analyzed under actual production conditions using the multi-zone reaction theory. Then, a comprehensive mechanism model for BOF steelmaking was established, and an integrated simulation of metallurgical reactions during the BOF steelmaking process was performed using FactSage thermodynamic software. Finally, the validity of this comprehensive model was verified through actual production data. The results show that the relative deviation of the cumulative decarburization rate ranges from −0.66% to 1.68%, while the absolute deviation of the calculated carbon content curve compared to the actual curve is less than 0.12%. This research helps clarify the variation patterns of key process parameters in BOF steelmaking, playing a significant role in advancing the intelligence of the BOF steelmaking process.

## 1. Introduction

BOF steelmaking is a complex, multi-variable, and multiphase high-temperature physicochemical reaction process characterized by rapid reaction rates and numerous influencing factors [1]. Achieving precise control of the steelmaking process solely through human experience is quite challenging. Typically, process analysis and simulation modeling are required to analyze the BOF steelmaking process, providing optimization solutions for BOF operations [2]. Therefore, in the context of carbon reduction and intelligent manufacturing in the steel industry, the analysis of metallurgical mechanisms and the development of control models for BOF steelmaking processes are crucial for enhancing control levels and advancing the intelligence of steelmaking.

For the simulation of the BOF steelmaking process, it is typically based on the establishment of a comprehensive mechanism model of the BOF. The modeling needs to analyze the main process behaviors of the BOF blowing process from the perspectives of metallurgical thermodynamics and kinetics to obtain key parameters for the model, thereby enabling the modeling and simulation of the BOF steelmaking process. Metallurgical scholars have conducted extensive research on modeling the BOF steelmaking process. For instance, the dynamic state of the BOF steelmaking process was analyzed based on unsteady-state mass transfer theory, establishing a comprehensive reaction mechanism model that considers processes such as lime slagging and scrap melting, simulating the changes in the composition of molten steel and slag [3,4]. However, in the fundamental assumptions of this model, the mass transfer of oxygen was always considered the limiting factor throughout the process, without accounting for the effects of changes in the mass transfer rates and states of various elements during different stages of smelting. Lytvynyuk et al. [5,6] developed a comprehensive model that fully couples thermodynamics and kinetics. However, this model treats the BOF as a homogeneous thermodynamic system with only a single reaction zone, neglecting the reactions between emulsified slag and metal droplets, as well as the impact of metal droplets on the slag–metal reaction interface.

With the continuous in-depth study of metallurgical mechanisms and the rapid development of simulation software, a new research approach has emerged: dividing the interior of the BOF into several relatively independent yet mutually coupled reaction zones based on the physical and chemical correspondence between metallurgical reactions and process phenomena, thereby establishing a comprehensive model based on multi-zone reaction mechanisms. Dogan et al. [7,8,9] divided the BOF steelmaking process into the molten bath reaction zone and emulsified phase reaction zone, establishing a comprehensive model for decarburization reactions from a kinetic perspective. In this model, the initial carbon content of droplets was equated to the carbon content in the molten bath phase, and the decarburization rate of droplets in the emulsified phase was calculated using the empirical model proposed by Brooks et al. [10]. Additionally, it assumed that decarburization in the molten bath only occurs at the gas–liquid interface in the jet impact zone, without considering the decarburization behavior at the slag–metal reaction on the surface of the molten bath, which has certain limitations. Furthermore, Dogan validated the model using data measured by Cicutti et al. [11,12] from a 200 t LD-LBE converter, which indicated that the amount of decarburization of droplets in the emulsified zone accounts for approximately 45% of the total decarburization in the BOF. The Dogan model treated decarburization reactions as the sole chemical reaction during BOF blowing, neglecting the oxidation of other elements and their effects on decarburization. To address this, Rout et al. [13,14,15,16,17,18] proposed a multi-zone reaction model coupled with a dynamic slag formation model based on the Dogan model. This model divides the BOF into three reaction zones: the jet impact zone, emulsified phase zone, and slag–metal body zone, incorporating a dynamic generation model of ${\mathrm{FeO}}_{n}$ and calculating the oxidation reactions of elements such as Si, Mn, and P separately. The decarburization rate is treated as a polynomial function dependent on the temperature and carbon content [19], while the oxidation of Si, Mn, and P is calculated based on the distribution ratio in the slag–metal balance [20,21]. Biswas et al. [22] developed a dynamic mixed-control model for BOF metal–slag–gas reactions to predict the evolution of the metal and slag compositions for the entire converter with the blowing time. Meanwhile, a decarburization model for a Fe–C droplet reacting in oxidizing slag was established, and the results showed that the model agrees well with the experimental data [23].

Sarkar et al. [24] established a dynamic prediction model for the composition of molten steel and slag during the BOF steelmaking process based on the principle of Gibbs free energy minimization (GEM). This model divides the BOF into emulsified phase reaction zones, upper molten bath reaction zones, and lower molten bath reaction zones. However, the overall reaction design of this model is relatively simple and does not consider the slag–metal reactions at the surface of the molten bath. When validated using data from Cicutti [11,12], it was found that the prediction of the reaction rate for Si and Mn was obviously too small. Kruskopf and Visuri [25] used the GEM method to model the BOF steelmaking reactions, dividing the BOF into three physical zones: the metallic phase body zone, slag phase body zone, and gas phase body zone, as well as a multiphase interface reaction zone. The equilibrium state of the reactions is calculated using the partition Gibbs energy (PGE) method [26]. Similarly, simulations and validations using data from Cicutti [11,12] revealed that the model’s predictions for the decarburization rate outperformed those of the Dogan and Sarkar models. However, the predicted silicon removal rate during the early reactions was significantly lower than the measured values, and the model did not consider the impact of secondary combustion reactions, indicating room for improvement. Van Ende and Jung et al. [27,28,29,30,31,32,33,34,35] proposed the concept of the effective equilibrium reaction zone (EERZ) and applied it to the simulation of the BOF steelmaking, secondary metallurgy (RH/LF), and continuous casting processes. The method integrates the kinetic factor and thermodynamic calculation and uses FactSage 7.0 thermodynamic calculation software to calculate the chemical equilibrium of each reaction zone in a step-by-step way to obtain the continuous change value of the molten metal phase and slag phase composition during the blowing process. Additionally, the model has been validated using plant data [11,12,32]. However, the EERZ parameters vary across different steelmaking plants and exhibit dynamic changes, necessitating that the modeling process be tailored to the specific operational conditions of each steelmaking plant.

Metallurgical researchers have made significant progress in studying BOF process models. The method of establishing comprehensive mechanism models based on multi-zone reaction theory has gained widespread application. Using thermodynamic software to simulate these models allows for both the demonstration of universal metallurgical principles and the flexibility to obtain model parameters under various operating conditions. This approach not only holds theoretical value from an academic perspective but has also been successfully validated using industrial data from several international steelmaking plants. However, the primary differences between the methods reported in the literature lie in the division of reaction zones within the BOF and the corresponding reaction parameters. Different reaction zones typically represent distinct metallurgical behaviors. Therefore, to establish an effective comprehensive model, a thorough analysis of the various process behaviors and their associated reaction parameters in BOF steelmaking is necessary.

This study integrates the actual BOF steelmaking process, firstly, based on the multi-zone reaction theory of comprehensive modeling, the BOF was divided into several reaction zones corresponding to key physical and chemical phenomena. Then, the physical and chemical behaviors of each reaction zone under actual production conditions were analyzed to obtain the corresponding thermodynamic and kinetic parameters. Finally, a comprehensive mechanism model of the BOF was established based on the analysis of the physical and chemical behaviors in each reaction zone. This model was coupled and computed using the Equilib module and Macro Processing function of the FactSage thermodynamic software to achieve an integrated simulation of the metallurgical reactions in the BOF steelmaking process. This study is helpful to clarify the variation patterns of the model parameters during the blowing process and then optimize the related steelmaking process and lay a foundation for the realization of intelligent steelmaking production.

## 2. Multi-Zone Reaction Division of BOF Steelmaking

**Figure 1:**
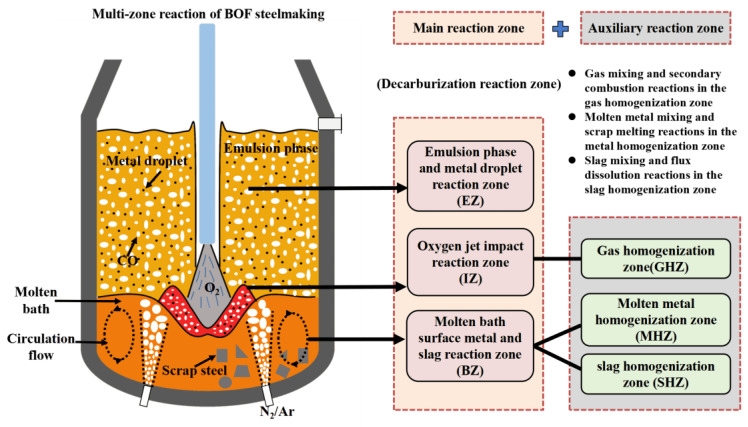
Multi-zone reaction division of BOF steelmaking.

In conjunction with the metallurgical reactions and process phenomena of the BOF steelmaking process, the multiphase reaction zones inside the BOF were divided into three main reaction zones and three auxiliary reaction zones. The three main reaction zones are the oxygen jet impact reaction zone (marked as IZ), the molten bath surface molten metal and slag reaction zone (marked as BZ), and the emulsion phase and metal droplet reaction zone (marked as EZ). The three auxiliary reaction zones are the gas homogenization zone (marked as GHZ), the molten metal homogenization zone (marked as MHZ), and the slag homogenization zone (marked as SHZ), as shown in Figure 1. Analyzing the metallurgical mechanisms of these reaction zones is the basis for developing the comprehensive BOF mechanism model. Therefore, the reaction mechanisms of these six zones are analyzed from both thermodynamic and kinetic perspectives in Section 3 and Section 4. The key metallurgical reactions discussed include the gas–liquid reactions in the jet impact zone, the slag–metal reactions in the molten bath surface zone, the metal droplets–slag reactions in the emulsion zone, the gas mixing and secondary combustion reactions in the gas homogenization zone, the molten metal mixing and scrap melting reactions in the molten metal homogenization zone, and the slag mixing and flux dissolution reactions in the slag homogenization zone.

## 3. Analysis of the Main Reaction Zones in BOF Steelmaking

### 3.1. Analysis of the Gas–Liquid Reactions in the Jet Impact Zone

In the jet impact zone, oxygen directly contacts the molten metal, initiating chemical reactions. This section primarily analyzes the gas–liquid reactions in the impact zone from both thermodynamic and kinetic perspectives.

#### 3.1.1. Thermodynamics of the Gas–Liquid Reactions

**Figure 2:**
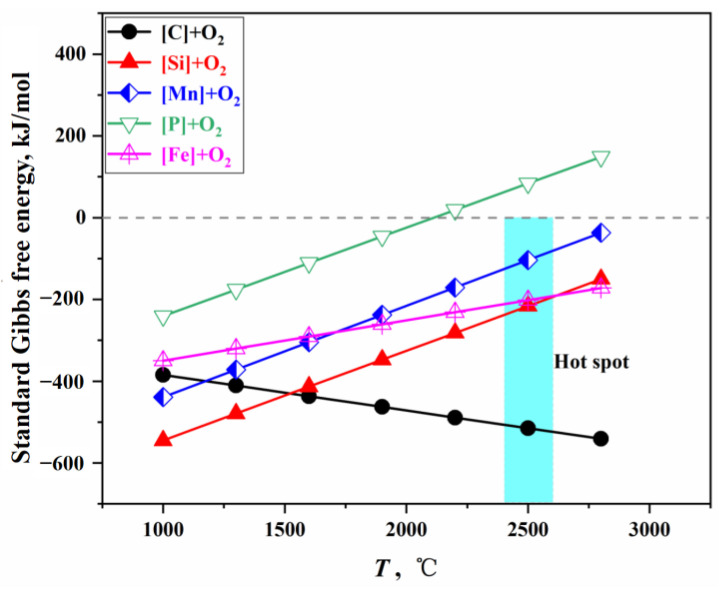
Gibbs free energy of direct oxidation reactions.

Elements such as C, Si, Mn, and Fe in the molten metal react directly with oxygen at the surface of the impact pit, undergoing intense oxidation reactions that release a significant amount of heat, resulting in an average surface temperature in the impact zone reaching approximately 2400 to 2600 °C; therefore, this region is referred to as the hot spot [36]. According to Section S1.1 in the Supplementary Materials, the standard Gibbs free energy changes for the oxidation reactions of C, Si, Mn, and Fe can be obtained, as shown in Figure 2. At this hot spot temperature, the $\Delta G^{\Theta }$ for P is greater than 0; therefore, it is concluded that no dephosphorization reaction occurs at the gas–liquid interface of the impact pit, and only the oxidation reactions of C, Si, Mn, and Fe take place.

**Figure 3:**
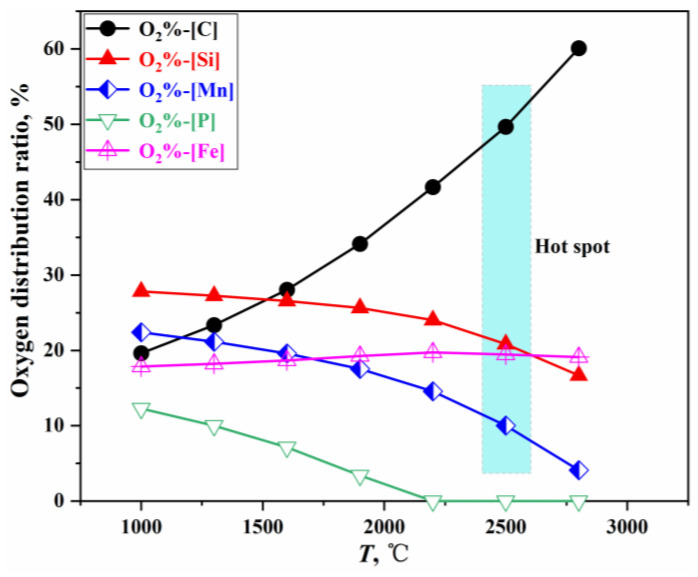
Oxygen distribution ratio of each element in a direct oxidation reaction.

When multiple elements coexist in the molten bath, the oxidation strength of each element is related to the oxygen potential for forming its oxide and the element’s activity. At equilibrium, the oxygen potentials of all oxides are equal [37]. By integrating the calculation methods for oxygen distribution ratios in the steelmaking process [38,39,40,41], and applying the PGE and the GEM [25,26], the relationship between the oxygen distribution ratios for each element and Gibbs free energy is established. The oxygen distribution ratios under standard conditions are illustrated in Figure 3, with a detailed calculation process provided in Section S1.2 of the Supplementary Materials.

#### 3.1.2. Dynamics Analysis of the Impact Zone

From a kinetic perspective, the impact pits generated by high-velocity jets exhibit high speed flow at the gas–liquid interface, rendering the traditional Higbie penetration theory not fully applicable. Due to the impact of gas flow erosion, the surface of the impact pit is not static but is continually refreshed, drawing molten steel from the molten bath to become a new reaction interface; this interface mass transfer is significantly influenced by the surface renewal rate [42]. The rapid renewal rate of the impact pit surface during high-velocity jet impacts results in mass transfer rates at the interface that are significantly higher than conventional transfer rates. Therefore, it is assumed that the oxygen reaching the impact pit surface and the molten steel flowing over it (i.e., the steel displaced by the oxygen jet) completely participate in the reaction. The reaction rates of oxidation in the impact zone can be calculated using the oxygen distribution ratios derived from gas–liquid reaction thermodynamics. The quantities of chemical reactions in the oxygen jet impact zone and their impact on the composition changes of the molten metal and slag are detailed in Section S1.3 of the Supplementary Materials.

### 3.2. Analysis of the Slag–Metal Reaction at the Molten Bath Surface Zone

In the products of the gas–liquid reactions within the jet impact zone, the carbon oxidation products, CO and CO_2_, are released from the furnace as part of the off-gas, while the oxidation products of other elements, such as FeO, MnO, and SiO_2_, primarily enter the slag phase above the molten metal bath. Oxidation reduction reactions occur between the molten metal and the slag at the surface of the molten bath.

#### 3.2.1. Thermodynamics of the Slag–Metal Reactions

During the BOF steelmaking process, the temperature in the jet impact zone is extremely high, with direct oxidation of elements such as C, Si, and Mn by oxygen being the dominant reactions, leading to the formation of a significant amount of FeO. However, at the slag–metal interface on the molten bath surface, indirect oxidation reactions primarily occur with FeO acting as the oxidizing agent. Given that chemical reactions at high temperatures tend to proceed rapidly, the following assumptions are made: (1) Reactions at the slag–metal interface reach equilibrium, and (2) the mass transfer rates of the components at the slag or molten metal side of the interface limit the reaction rate. Using the mass transfer rate of reactants on the molten metal side, the mass transfer rate of products on the slag side, and the activities of the steel and slag components, the equilibrium concentrations of the components at the slag–metal interface are obtained through an iterative method. Subsequently, the changes in the composition of the steel and slag due to the interface reactions are calculated. The detailed calculation process can be found in Section S2.1 of the Supplementary Materials and Ref. [43].

#### 3.2.2. Dynamics Analysis of the Molten Bath Surface Zone

**Figure 4:**
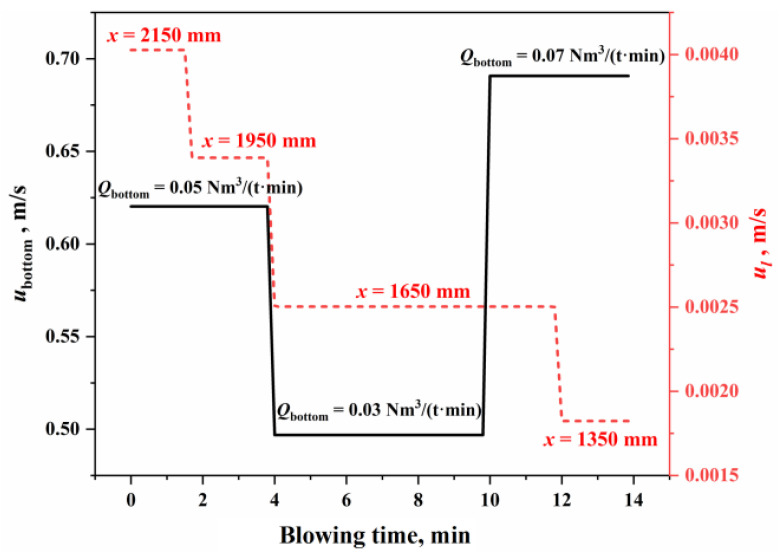
Circulation renewal speed of the slag–metal interface.

From a kinetic perspective, the slag–metal interface on the molten bath surface is influenced by the combined effects of top and bottom blowing, where the circulation renewal speed at the interface ($u_{\mathrm{sm}}$) is the comprehensive impacts of top blowing ($u_{l}$) and bottom blowing ($u_{\mathrm{bottom}}$). Figure 4 shows the circulation renewal speed of the slag–metal interface under typical operating conditions in this study. The calculation process for $u_{l}$ and $u_{\mathrm{bottom}}$ is detailed in Section S2.2 of the Supplementary Materials and Refs. [44,45]. As shown in Figure 4, the circulation renewal speed caused by top blowing is two orders of magnitude lower than that caused by bottom blowing. Therefore, the kinetic conditions at the slag–metal interface on the molten bath surface are primarily controlled by bottom blowing, with top blowing having a relatively smaller impact. Under typical operating conditions in this study, the circulation renewal speed of the slag–metal interface on the molten bath surface is 0.5–0.69 m/s.

**Figure 5:**
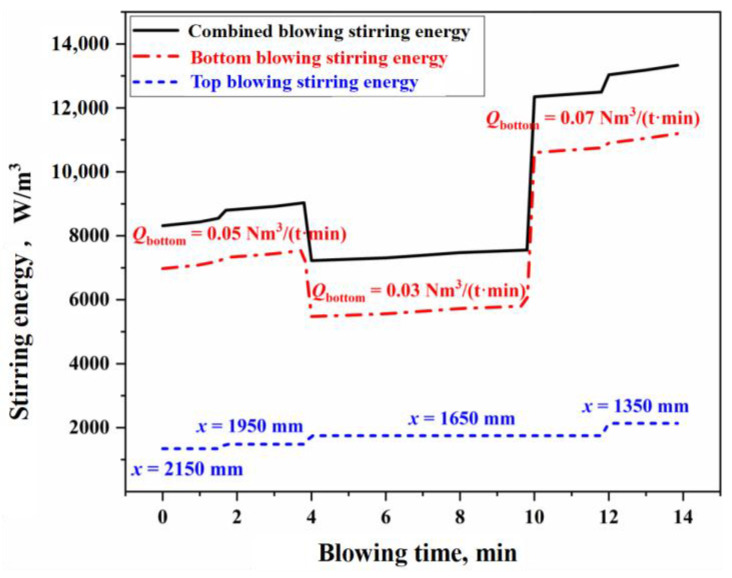
Variation of combined stirring energy in the BOF steelmaking process.

Figure 5 illustrates the variation of combined stirring energy in the BOF during combined blowing under typical operating conditions in this study. The calculation process is detailed in Section S2.3 of the Supplementary Materials. In Figure 5, the bottom blowing stirring energy ($\varepsilon_{B}$) contributes approximately 80% to the total combined stirring energy ($\varepsilon_{\mathrm{tot}}$), while the impact of the top blowing jet on the overall stirring energy is relatively smaller. This indicates that the stirring intensity of the molten bath is primarily determined by the bottom blowing strength. Consequently, the slag/molten metal mass transfer rate at the molten bath surface is also mainly governed by bottom blowing, which is consistent with the conclusions drawn earlier from the analysis of the slag–metal circulation renewal speed.

**Figure 6:**
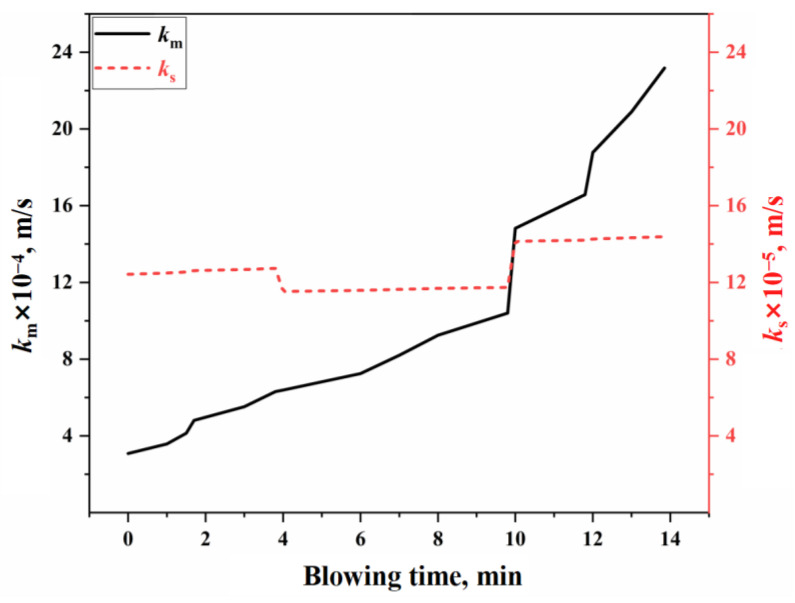
Variation in effective mass transfer coefficient of slag and molten metal.

Based on the reaction volume method of the effective equilibrium reaction theory, it is assumed that the mass transfer rates of all components in the bulk phase within the effective equilibrium reaction zone are identical [37], allowing for the calculation of the mass transfer coefficient on the molten metal side ($k_{m}$) and the slag side ($k_{s}$) [5,46]. Figure 6 illustrates the variation in effective mass transfer coefficient of slag and molten metal on the surface of the molten bath during the blowing process under typical operating conditions in this study. The calculation process is provided in Section S2.3 of the Supplementary Materials. As shown in Figure 6, the effective mass transfer coefficient for the molten metal side is (3.08–23.18) × 10^−4^ m/s, while the coefficient for the slag side is (12.42–14.38) × 10^−5^ m/s. Using these results, along with the surface area parameters of the molten bath, the effective reaction volume participating in the slag–metal reaction at the molten bath surface can be determined for use in molten bath surface process reaction modeling.

### 3.3. Analysis of the Metal Droplet Reaction in the Emulsified Zone

Similar to the slag–metal reaction occurring at the surface of the molten bath, the reactions involving metal droplets in the emulsion zone are also fundamentally slag–metal reactions. The basic thermodynamic principles have already been explained in the previous section. However, the key difference lies in the kinetic conditions, which are altered by the droplet expansion and flight behaviors. As a result, it is necessary to conduct an in-depth analysis and justification of the relevant kinetic factors.

#### 3.3.1. Dynamics of the Decarburization Reactions of Metal Droplets

For carbon-containing metal droplets, oxidation and decarburization will occur upon entering the slag. The carbon content of the droplets, the FeO content in the slag, and the droplet diameter are the primary influencing factors. This also indicates that the decarburization behavior of the droplets is closely related to these three factors [47]. The decarburization rate expression, which correlates with the variables of droplet carbon content ($w_{\left[C\right]}^{b}$), FeO content in slag ($w_{\left(\mathrm{FeO}\right)}^{b}$), and droplet diameter (*d*), is shown in Equation (1), with the derivation process provided in Section S3.1 of the Supplementary Materials. As indicated by Equation (1), the instantaneous decarburization rate of the droplets is not only influenced by the three main factors ($w_{\left[C\right]}^{b}$, $w_{\left(\mathrm{FeO}\right)}^{b}$, and *d*) but is also dependent on the instantaneous velocity of the droplets ($u_{d}$).

(1) $$\frac{dw_{\left[C\right]}}{dt}=\left\{\begin{array}{l} \;\frac{1}{50}\times \sqrt{\frac{D_{\mathrm{FeO}}u_{d}}{\pi d^{3}}}\times {\left(1-\frac{f_{\mathrm{solid}}}{f_{\mathrm{solid}}^{*}}\right)}^{1.25}\times {\left(1-f_{\mathrm{solid}}\right)}^{2/3}\times \frac{\rho_{s}}{\rho_{d}}\times \left[w_{\left(\mathrm{FeO}\right)}^{b}-\frac{1}{96.6f_{C}w_{\left[C\right]}^{b}}\times \frac{M_{\mathrm{FeO}}}{M_{\mathrm{slag}}}\right],\;\;\left(J_{\mathrm{FeO}}/J_{C}\right)\leq 1\\ \;\frac{3}{25}\times \sqrt{\frac{D_{C}u_{d}}{\pi d^{3}}}\times {\left(1-f_{\mathrm{solid}}\right)}^{2/3}\times \left(w_{\left[C\right]}^{b}-\frac{1}{96.6\gamma_{\left(\mathrm{FeO}\right)}w_{\left(\mathrm{FeO}\right)}^{b}}\times \frac{M_{\mathrm{FeO}}}{M_{\mathrm{slag}}}\right)\;\;\;\;\;\;\;\;,\;\;\left(J_{\mathrm{FeO}}/J_{C}\right)>1 \end{array}\right.$$

#### 3.3.2. Effective Reaction Amount of Molten Metal in the Emulsified Zone

Using the decarburization rate calculation formula for the droplets shown in Equation (1), the decarburization amount of a single droplet in the emulsified slag can be determined. By combining this with the droplet generation rate and its particle size distribution (the calculation process can be found in Section S3.2 of the Supplementary Materials and Refs. [48,49,50,51]), the total decarburization amount of all metal droplets reacting in the emulsified slag during the actual blowing process in the BOF can be calculated, thereby determining the effective reaction amount of the slag–metal reaction in the emulsified zone.

According to the droplet size distribution, the overall effective decarburization rate of all droplets ($\Delta C_{d}^{\mathrm{tot}}$) can be obtained through the integral expression, as shown in Equation (2):

(2) $$\Delta C_{d}^{\mathrm{tot}}=\int_{0}^{d_{\mathrm{limit}}}f\left(d\right)\times \Delta C_{d}\times d\left(d\right)$$

where $f\left(d\right)$ is the proportion of droplets of different diameters,%; $d_{\mathrm{limit}}$ is the maximum diameter of the droplet, mm; and $\Delta C_{d}$ is the effective decarburization rate of a single droplet, with a value ranging from 0 to 1, as derived from Equation (S77) in Section S3.3 of the Supplementary Materials.

**Figure 7:**
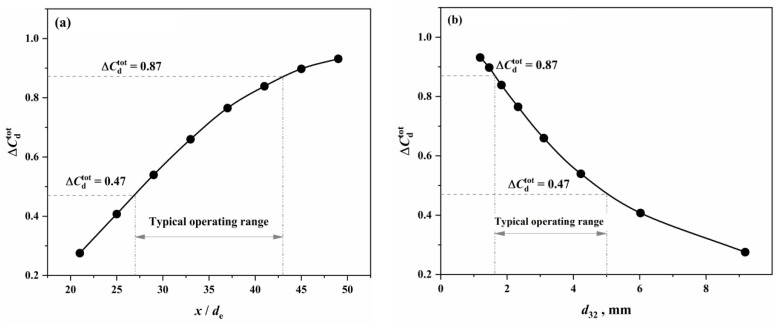
Overall effective decarburization rate of all droplets under different (**a**) oxygen lance positions and a (**b**) characteristic reaction diameter.

According to Equation (2), the $\Delta C_{d}^{\mathrm{tot}}$ under the conditions of this study was calculated using the dimensionless oxygen lance position ($x/d_{e}$) and the characteristic reaction diameter ($d_{32}$) as the horizontal coordinates, as shown in Figure 7.

From Figure 7, it can be observed that, as the operational oxygen lance position decreases (resulting in larger droplet diameters) and the characteristic reaction diameter increases, the comprehensive effective decarburization rate of the metal droplets decreases. This indicates that the reaction performance of the droplets declines with the increasing diameter, and there is a good correlation between the characteristic reaction diameter and the reaction performance of the droplets. Under the conditions of this study, the typical operational oxygen lance position range of 1350 to 1950 mm corresponds to a comprehensive effective decarburization rate of 0.47 to 0.87 for the metal droplets.

Since the decarburization behavior is integral to the entire reaction process between the droplets and the emulsified slag, and the kinetic factors of the reaction are primarily influenced by the expansion behavior resulting from decarburization, the comprehensive effective decarburization rate of the Fe–C droplets can be used to approximate the effective reaction proportion of the metal droplets (i.e., the ratio of the effective equilibrium reaction amount of the droplets to the total amount of droplets, $PCT_{d}^{\mathrm{EERZ}}$). Therefore, the effective equilibrium reaction amount of the metal droplets in the emulsified phase can be expressed as Equation (3):

(3) $$W_{d}^{\mathrm{EERZ}}=R_{B,T}\cdot PCT_{d}^{\mathrm{EERZ}}=R_{B,T}\cdot \Delta C_{d}^{\mathrm{tot}}$$

where $R_{B,T}$ is the amount of droplet generated per unit time, kg/s; $PCT_{d}^{\mathrm{EERZ}}$ is the ratio of the effective equilibrium reaction amount of the droplets to the total amount of droplets, %; and $W_{d}^{\mathrm{EERZ}}$ is the effective equilibrium reaction amount of the droplet, kg/s.

From Equation (3), the effective equilibrium reaction amount of the droplet in the emulsified phase can be determined. By calculating the chemical reaction equilibrium, the reaction amounts of various elements in the emulsified reaction zone can be obtained, along with their impact on the compositions of the molten metal and slag.

## 4. Analysis of the Auxiliary Reaction Zones in BOF Steelmaking

To establish a comprehensive model for the BOF mechanism, it is essential to analyze not only the reaction behaviors in the main reaction zones but also the metallurgical reactions in the auxiliary reaction zones. Among the three auxiliary reaction zones, in addition to the physical mixing behavior of the gas, slag, and molten metal phases, the gas mixing zone primarily involves secondary combustion reactions of the furnace gas, the molten metal mixing zone mainly involves the melting reactions of scrap steel, and the slag mixing zone primarily involves the dissolution reactions of CaO and MgO fluxes.

### 4.1. Analysis of the Secondary Combustion Reaction in the Gas Phase Mixing Zone

In the jet impact zone, the gaseous products generated from the gas–liquid reactions occurring at the surface of the impact crater are referred to as the primary furnace gas. After the primary furnace gas escapes from the surface of the impact crater, it undergoes secondary combustion reactions with the oxygen present at the edge of the jet. Assuming the total oxygen supply per unit time is $W_{O_{2}}^{\mathrm{tot}}$, the relationship between the oxygen consumed in the gas–liquid reaction in impact zone $W_{O_{2}}^{\mathrm{IZ}}$ and the oxygen consumed in the secondary combustion $W_{O_{2}}^{\mathrm{GHZ}}$ is described by Equation (4):

(4) $$W_{O_{2}}^{\mathrm{tot}}=W_{O_{2}}^{\mathrm{IZ}}+W_{O_{2}}^{\mathrm{GHZ}}$$

For top-blown oxygen converters, the theoretical secondary combustion rate can be calculated using Equation (5) [52]:

(5) $$PCR=0.1\times {\left(\frac{x-x_{1}}{d_{\mathrm{th}}}\right)}^{0.3}-{\left(\frac{x-x_{1}}{d_{\mathrm{th}}}\right)}^{-0.7}+0.01$$

where x is the oxygen lance position, m; $x_{1}$ is the jet core section length, m; and $d_{\mathrm{th}}$ is the oxygen lance nozzle throat diameter, m.

**Figure 8:**
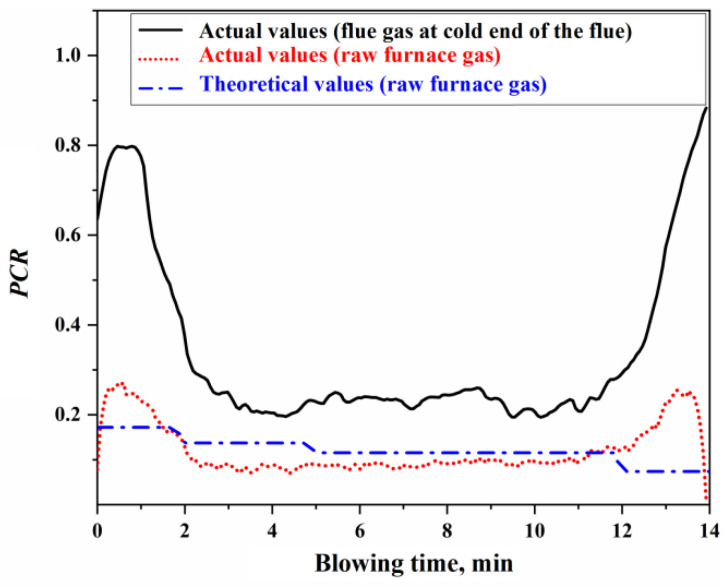
Actual and theoretical secondary combustion rates.

Based on the flue gas composition data from actual production, the actual values for the secondary combustion rate at the cold end of the flue and the secondary combustion rate of the raw furnace gas within the BOF can be determined. The calculation process is detailed in Section S4 of the Supplementary Materials. Figure 8 presents the results of the actual and theoretical secondary combustion rates.

**Figure 9:**
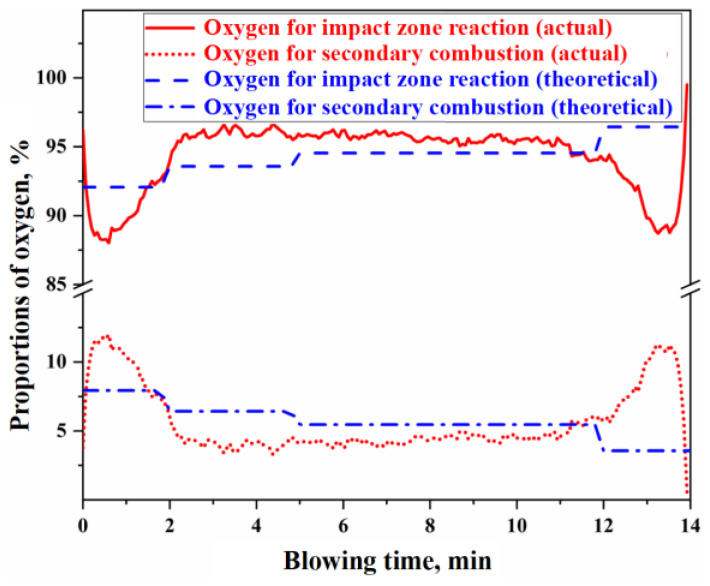
Proportion of oxygen used for the impact zone reaction and secondary combustion.

Using the actual or theoretical values of the secondary combustion rate of the raw furnace gas shown in Figure 8, the proportions of oxygen in the top-blown jet used for the direct oxidation reaction in the impact zone and the secondary combustion reaction of the furnace gas can be determined, as illustrated in Figure 9. The calculation process is detailed in Section S4 of the Supplementary Materials. From Figure 9, it can be concluded that the ratio of oxygen in the top-blown jet, which is used for both the direct oxidation reaction in the impact zone and the secondary combustion reaction of the furnace gas, is approximately 94:6, indicating that about 6% of the oxygen in the top-blown air is utilized for the secondary combustion of the furnace gas.

From Figure 8 and Figure 9, it can be observed that there is a significant discrepancy between the theoretical and actual values of the secondary combustion rate during the initial 2 min and the final 2 min of the blowing process. This discrepancy is primarily attributed to the limited volume of raw furnace gas generated during the initial and final stages of the decarburization reaction, resulting in the actual secondary combustion rate being higher than the theoretical value. To mitigate the computational bias caused by this factor during metallurgical reaction calculations, the actual PCR curve can be utilized. When the $W_{O_{2}}^{\mathrm{tot}}$ is known, the specific values of $W_{O_{2}}^{\mathrm{IZ}}$ and $W_{O_{2}}^{\mathrm{GHZ}}$ can be derived based on the oxygen used ratio of the impact zone reaction and the secondary combustion reaction. These values can then be substituted into the calculations for the gas–liquid reactions in the impact zone and the secondary combustion reactions in the gas phase to obtain the corresponding reaction results.

### 4.2. Analysis of the Scrap Steel Melting Reaction in the Molten Metal Mixing Zone

**Figure 10:**
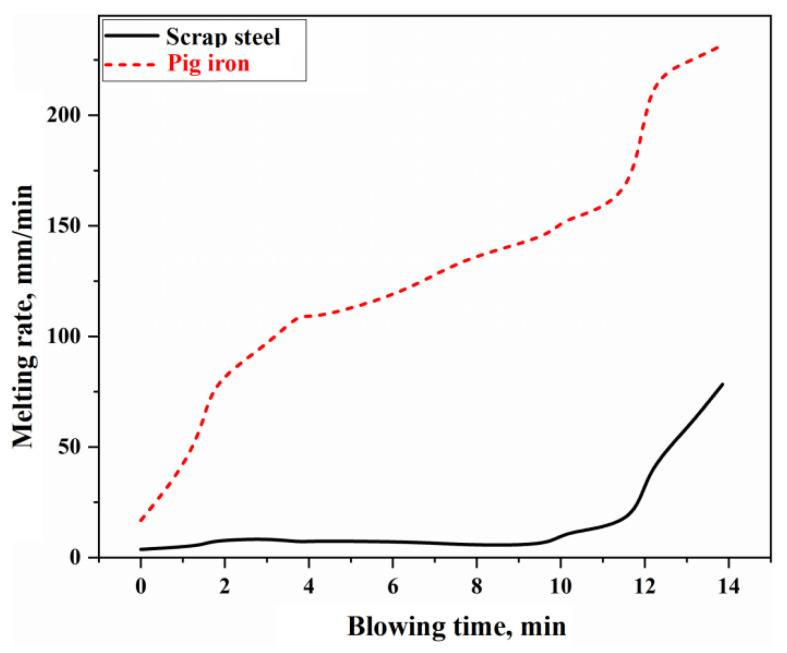
Melting rate of scrap steel and pig iron.

As the most crucial reaction in the molten metal mixing zone of the BOF, the melting of scrap steel in the molten bath primarily occurs through two mechanisms: carbon concentration-driven melting and thermally driven melting [5]. The stirring intensity of the molten bath directly affects the mass transfer and heat transfer rates of the molten metal, which, in turn, influences the rate of scrap steel melting. By substituting the stirring energy of the molten bath under the typical working conditions of this study, as illustrated in Figure 5, into Equations (S59) and (S88) in the Supplementary Materials, the mass transfer coefficient $k_{m}$ and heat transfer coefficient $h_{m}$ can be determined. Consequently, the melting rate curves for scrap steel and pig iron during the BOF steelmaking process can be obtained, as shown in Figure 10. The melting of scrap steel is influenced by both carbon concentration and thermal driving forces. Its melting rate is derived through a single-variable solution of the simultaneous equations in the Supplementary Materials, Equations (S86) and (S87). In contrast, pig iron, due to its high carbon content and the molten bath temperature consistently exceeding the melting point of pig iron, is predominantly controlled by thermal driving mechanisms, with its melting rate calculated using Equation (S87) from the Supplementary Materials.

In Figure 10, the melting rate of pig iron is relatively fast, with complete melting occurring approximately 2 to 3 min after the BOF starts blowing. However, the melting rate of scrap steel is slower, resulting in a longer melting duration. During the latter stages of the blowing process (after about 12 min), the melting rate of scrap steel significantly increases due to the enhanced bottom blowing intensity and elevated molten bath temperatures.

### 4.3. Analysis of the Flux Dissolution Reaction in the Slag Homogenization Zone

**Figure 11:**
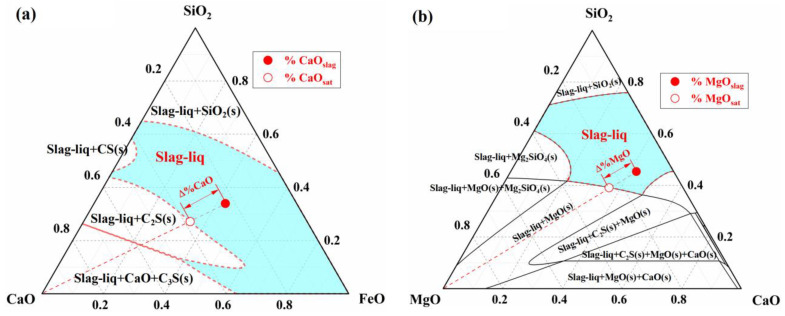
Dissolution driving force of (**a**) the CaO in CaO-SiO_2_-FeO slag system and (**b**) the MgO in MgO-CaO-SiO_2_-20%FeO slag system.

In the slag homogenization zone, the dissolution of CaO and MgO fluxes into the slag is primarily driven by the concentration difference between the actual concentrations of CaO and MgO in the slag and their respective saturation concentrations. According to the dissolution rate equations of CaO and MgO fluxes in the slag (refer to Section S6 of the Supplementary Materials and Refs. [53,54]), the dissolution rate is jointly determined by the saturation concentration difference and the effective mass transfer coefficient in the slag. The saturation concentrations of CaO and MgO in the slag are obtained through phase diagram calculations. For example, at 1773 K, the phase diagrams of the CaO-SiO_2_-FeO slag system and the MgO-CaO-SiO_2_-20%FeO slag system, along with the driving force for flux dissolution, are shown in Figure 11.

**Figure 12:**
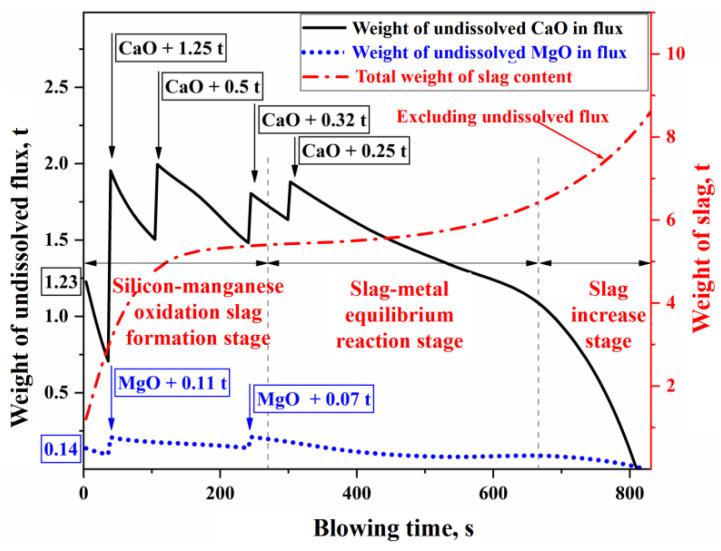
Dissolution process of CaO and MgO fluxes in the blowing process of Heat A.

In Figure 11a, the solid–liquid coexistence zone between the liquid phase zone and the pure CaO component represents the precipitation region of solid phases such as CaO, $2\mathrm{CaO}\cdot {\mathrm{SiO}}_{2}$ (C2S), and $3\mathrm{CaO}\cdot {\mathrm{SiO}}_{2}$ (C3S). The intersection of the line connecting the pure CaO component and the actual composition point ($\%{\mathrm{CaO}}_{\mathrm{slag}}$) (represented by the dashed line in Figure 11a) with the solid phase precipitation boundary defines the saturation concentration of CaO ($\%{\mathrm{CaO}}_{\mathrm{sat}}$). This allows for the determination of the concentration difference between the saturation and actual concentrations (Δ%CaO), which serves as the driving force for the dissolution reaction of CaO flux. Similarly, the saturation concentration ($\%{\mathrm{MgO}}_{\mathrm{sat}}$) and concentration difference (Δ%MgO) for MgO are obtained using the same method in Figure 11b. By utilizing the phase diagram calculation module and macro programming functions of FactSage thermodynamic software, the dissolution behavior of fluxes in slag during the BOF blowing process was simulated. Taking experimental Heat A as an example, the results are shown in Figure 12.

In Figure 12, Heat A involved the batch addition of fluxes such as lime and dolomite, which contain CaO and MgO. The dissolution characteristics during the refining process are as follows: In the early stage of refining (the silicon–manganese oxidation slag formation stage), a large amount of silicon and manganese in the molten metal is oxidized into the slag, resulting in a significant concentration gradient driving force for the dissolution of CaO and MgO in the slag. This leads to a faster dissolution rate of the fluxes. In the middle stage of refining (the slag–metal equilibrium reaction stage), the oxidation of silicon and manganese is nearly complete, and rapid decarburization of the molten bath results in a low FeO content in the slag. This lowers the concentration gradient driving force for CaO and MgO dissolution, and with weak stirring from the bottom blowing at this stage, mass transfer within the slag slows down. Consequently, the dissolution rate of the fluxes and the overall slag volume change at a relatively steady pace during this period. In the later stage of refining (the slag increase stage), as the carbon content in the molten bath decreases, decarburization weakens, and a greater proportion of the top-blown oxygen is used for Fe oxidation. The increase in FeO content in the slag enhances the concentration gradient driving force, while the stronger stirring of the molten bath improves the effective mass transfer coefficient in the slag, resulting in a significantly accelerated dissolution of the fluxes. Finally, before the end of the refining process, the CaO and MgO in the fluxes are fully dissolved into the slag.

## 5. Comprehensive Simulation of Multi-Zone Reactions in BOF Steelmaking

The interfacial reaction model posits that multiphase reactions occur primarily at the interface between phases. However, the EERZ theory suggests that, on both sides of the phase boundary, there exists an effective diffusion zone for reactants and products (EERZ), where reactions occur and reach thermodynamic equilibrium rather than being confined solely to the interface between the two phases [35]. Based on the analysis of the multi-zone reaction mechanisms during BOF steelmaking, a comprehensive BOF mechanism model was developed considering multiple reaction zones. FactSage thermodynamic software was then used to perform coupled calculations for both the comprehensive model and the metallurgical reactions in each zone, thus enabling comprehensive simulations of the metallurgical reactions throughout the BOF steelmaking process.

### 5.1. Establishment of a Comprehensive Model of the Multi-Region Reaction Mechanism

For the six effective reaction zones in BOF steelmaking, the balance relations of effective reaction amounts and the simplified expressions for the six reactions (R1~R6) are shown in Equations (6) and (7). R1~R6 correspond to the reactions in the oxygen jet impact reaction zone (IZ), molten bath surface molten metal and slag reaction zone (BZ), emulsion and metal droplet reaction zone (EZ), gas homogenization zone (GHZ), molten metal homogenization zone (MHZ), and slag homogenization zone (SHZ).

(6) $$\left\{\begin{array}{l} W_{\mathrm{metal}}^{\mathrm{tot}}=W_{\mathrm{metal}}^{\mathrm{BZ}}+W_{\mathrm{metal}}^{\mathrm{IZ}}+W_{\mathrm{metal}}^{\mathrm{MHZ}}+W_{\mathrm{scrap}}\\ W_{\mathrm{slag}}^{\mathrm{tot}}=W_{\mathrm{slag}}^{\mathrm{BZ}}+W_{\mathrm{slag}}^{\mathrm{EZ}}+W_{\mathrm{slag}}^{\mathrm{SHZ}}+W_{\mathrm{flux}}\\ W_{O_{2}}^{\mathrm{tot}}=W_{O_{2}}^{\mathrm{IZ}}+W_{O_{2}}^{\mathrm{GHZ}} \end{array}\right\}$$

(7) $$\left\{\begin{array}{ll} R1: & W_{\mathrm{metal}}^{\mathrm{IZ}}+W_{O_{2}}^{\mathrm{IZ}}\to {\left(W_{\mathrm{metal}}^{\mathrm{IZ}}\right)}^{\prime }+W_{\mathrm{slag}}^{\mathrm{IZ}}+W_{\mathrm{gas}}^{\mathrm{IZ}}\\ R2: & W_{\mathrm{metal}}^{\mathrm{BZ}}+W_{\mathrm{slag}}^{\mathrm{BZ}}\to {\left(W_{\mathrm{metal}}^{\mathrm{BZ}}\right)}^{\prime }+{\left(W_{\mathrm{slag}}^{\mathrm{BZ}}\right)}^{\prime }+W_{\mathrm{gas}}^{\mathrm{BZ}}\\ R3: & W_{\mathrm{droplet}}^{\mathrm{EZ}}+W_{\mathrm{slag}}^{\mathrm{EZ}}\to {\left(W_{\mathrm{droplet}}^{\mathrm{EZ}}\right)}^{\prime }+{\left(W_{\mathrm{slag}}^{\mathrm{EZ}}\right)}^{\prime }+W_{\mathrm{gas}}^{\mathrm{EZ}}\\ R4: & W_{\mathrm{gas}}^{\mathrm{IZ}}+W_{O_{2}}^{\mathrm{GHZ}}+W_{\mathrm{gas}}^{\mathrm{BZ}}+W_{\mathrm{gas}}^{\mathrm{EZ}}\to W_{\mathrm{gas}}^{\mathrm{tot}}\\ R5: & \left[{\left(W_{\mathrm{metal}}^{\mathrm{IZ}}\right)}^{\prime }-W_{\mathrm{droplet}}^{\mathrm{EZ}}+{\left(W_{\mathrm{droplet}}^{\mathrm{EZ}}\right)}^{\prime }\right]+{\left(W_{\mathrm{metal}}^{\mathrm{BZ}}\right)}^{\prime }+W_{\mathrm{metal}}^{\mathrm{MHZ}}+W_{\mathrm{scrap}}\to W_{\mathrm{metal}}^{\mathrm{tot}}\\ R6: & W_{\mathrm{slag}}^{\mathrm{IZ}}+{\left(W_{\mathrm{slag}}^{\mathrm{BZ}}\right)}^{\prime }+{\left(W_{\mathrm{slag}}^{\mathrm{EZ}}\right)}^{\prime }+W_{\mathrm{slag}}^{\mathrm{SHZ}}+W_{\mathrm{flux}}\to W_{\mathrm{slag}}^{\mathrm{tot}} \end{array}\right\}$$

where $W_{\mathrm{metal}}^{\mathrm{tot}}$, $W_{\mathrm{slag}}^{\mathrm{tot}}$, $W_{O_{2}}^{\mathrm{tot}}$, and $W_{\mathrm{gas}}^{\mathrm{tot}}$ are the total amount of molten metal, slag, oxygen, and furnace gas, respectively, kg; $W_{\mathrm{metal}}^{\mathrm{IZ}}$ and $W_{O_{2}}^{\mathrm{IZ}}$ are the amount of molten metal and oxygen in the jet impact zone participating in the effective equilibrium reaction, respectively, kg; ${\left(W_{\mathrm{metal}}^{\mathrm{IZ}}\right)}^{\prime }$, $W_{\mathrm{slag}}^{\mathrm{IZ}}$, and $W_{\mathrm{gas}}^{\mathrm{IZ}}$ are the amount of molten metal, slag, and furnace gas in the jet impact zone that effectively balances in the reaction product, respectively, kg; $W_{\mathrm{metal}}^{\mathrm{BZ}}$ and $W_{\mathrm{slag}}^{\mathrm{BZ}}$ are the amount of molten metal and molten slag on the surface of the molten bath participating in the effective equilibrium reaction, respectively, kg; ${\left(W_{\mathrm{metal}}^{\mathrm{BZ}}\right)}^{\prime }$, ${\left(W_{\mathrm{slag}}^{\mathrm{BZ}}\right)}^{\prime }$, and $W_{\mathrm{gas}}^{\mathrm{BZ}}$ are the amount of molten metal, slag, and furnace gas in the product of the effective equilibrium reaction on the surface of the molten bath, respectively, kg; $W_{O_{2}}^{\mathrm{GHZ}}$ is the amount of oxygen in the gas mixed zone participating in the secondary combustion reaction, kg; $W_{\mathrm{droplet}}^{\mathrm{EZ}}$, $W_{\mathrm{slag}}^{\mathrm{EZ}}$, and $W_{\mathrm{gas}}^{\mathrm{EZ}}$ are the amount of metal droplet, slag, and furnace gas in the emulsion zone participating in the effective equilibrium reaction, respectively, kg; ${\left(W_{\mathrm{droplet}}^{\mathrm{EZ}}\right)}^{\prime }$, ${\left(W_{\mathrm{slag}}^{\mathrm{EZ}}\right)}^{\prime }$, and ${\left(W_{\mathrm{gas}}^{\mathrm{EZ}}\right)}^{\prime }$ are the amount of molten metal, molten slag, and furnace gas in the product of effective equilibrium reaction in the emulsion zone of slag–metal, respectively, kg; $W_{\mathrm{metal}}^{\mathrm{MHZ}}$ is the amount of molten metal in the molten metal mixing zone only participating in physical mixing, kg; $W_{\mathrm{slag}}^{\mathrm{SHZ}}$ is the amount of slag in the slag mixing zone only participating in physical mixing, kg; and $W_{\mathrm{scrap}}$ and $W_{\mathrm{flux}}$ are the melting amount of scrap steel and the dissolving amount of flux, respectively, kg.

Based on the analysis of the six reaction zones in this study and fundamental metallurgical principles, such as Gibbs free energy minimization, the equilibrium of the metallurgical reactions in each zone is computed at a certain time step. This process results in a comprehensive model for the multi-zone reaction mechanism in the BOF process, integrating both thermodynamic and kinetic factors.

### 5.2. Discussion on the Simulation Results of the BOF Steelmaking Process

According to the formatting requirements of FactSage software, the corresponding macro code was written. Subsequently, the macro code file was executed using the Macro Processing function of the software to perform calculations and coupling of the multi-zone reactions R1 to R6 in Equation (7), iterating until the oxygen blowing process was completed.

Taking experimental Heat A as an example, the related parameters are presented in Table 1. The total oxygen blowing time for this heat was 831 s, with a time step of 5 s, resulting in a total of 166 iterations. The total simulation time was 139 min.

**Figure 13:**
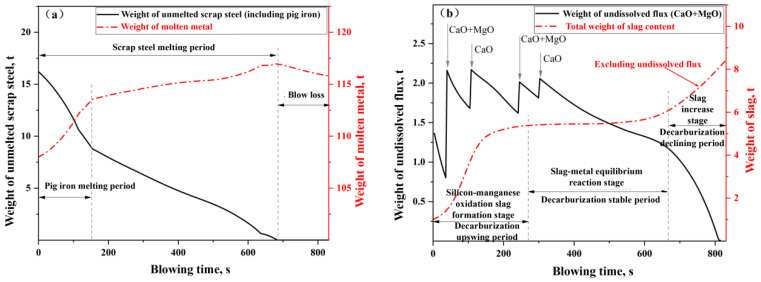
Weight change of (**a**) molten metal and (**b**) slag in the blowing process of Heat A.

**Figure 14:**
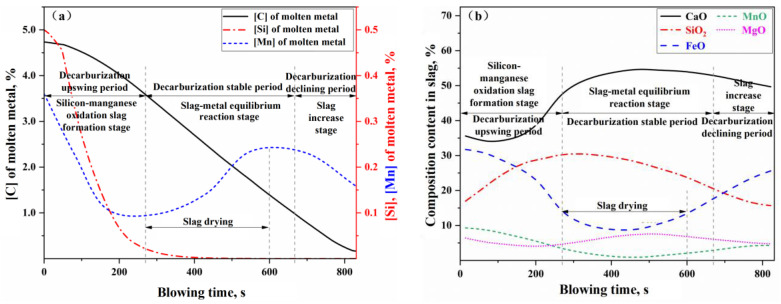
Component change of (**a**) molten metal and (**b**) slag in blowing process of Heat A.

**Figure 15:**
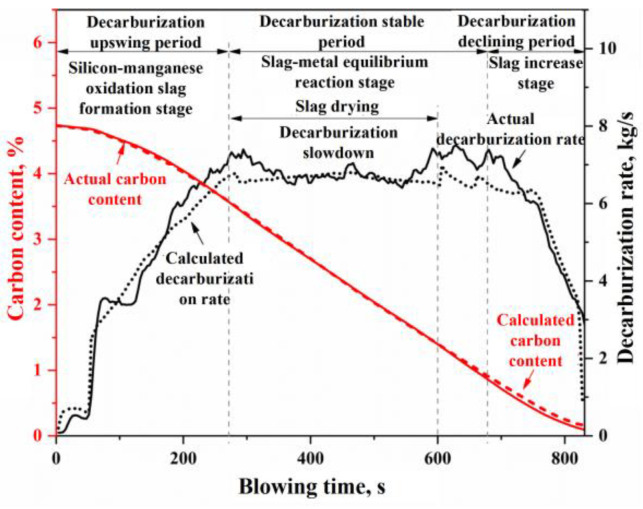
Changes in the decarburization rate and carbon content in the blowing process of Heat A.

The simulation results for Heat A are shown in Figure 13, Figure 14 and Figure 15. Figure 13a,b present the simulated results of the weight changes for the molten metal and slag phases during the blowing process of Heat A, respectively. Figure 14a,b illustrate the simulated results of the composition for the molten metal and slag phases, respectively. Figure 15 compares the simulated results of the decarburization rate and carbon content changes during the blowing process with the measured results. In Figure 15, the actual decarburization rate is calculated based on the flow and composition of the flue gas, while the actual carbon content change curve is derived from the raw input material information, the final molten metal chemical composition results, and the flue gas analysis data during the blowing process.

In Figure 13a, the weight change curve of the molten metal phase can be roughly divided into three stages: the pig iron melting stage, the scrap melting stage (excluding pig iron), and the net loss stage. Specifically, the process enters the pig iron melting stage immediately after the start of blowing, where the molten bath temperature is above the melting point of pig iron but below that of scrap steel. The pig iron blocks in the scrap heat rapidly melt due to thermal drive, and some of the scrap also melts due to carburizing effects, leading to a rapid increase in the weight of the molten metal, surpassing the oxidation and removal rates of elements in the molten metal (mainly the oxidation of silicon and manganese, along with some decarburization). After the pig iron blocks completely melt, the remaining scrap continues to melt. In the early to mid-stages of the scrap melting phase, the molten bath temperature is below the melting point of the scrap, and the melting of the scrap is primarily driven by carburizing, resulting in a relatively slow melting rate. However, the weight of the molten metal continues to increase at a rate that exceeds the oxidation and removal rates of its elements (mainly oxidation and decarburization), albeit more slowly. In the later stages of the scrap melting phase, the molten bath temperature exceeds the melting point of the scrap, leading to an accelerated melting process driven by heat, which, in turn, increases the weight of the molten metal. Once the scrap is fully melted, the process enters the net loss stage, during which the weight of the molten metal begins to decrease, primarily due to oxidation, decarburization, and iron loss, until the end of the blowing process.

In Figure 13b, the weight change curve of the slag phase can also be roughly divided into three stages: the oxidation of silicon and manganese into slag (or decarburization upswing period), the stable reaction period of slag and molten metal (or decarburization stable period), and the slag iron rising period (or decarburization declining period). After the blowing process begins (0–270 s), a significant amount of silicon and manganese oxidizes into the slag, resulting in a rapid dissolution of the added CaO and MgO flux, leading to a swift increase in the slag quantity within the furnace; this stage is characterized as the rapid slag formation period. Once silicon and manganese oxidize to a certain extent, the process enters the stable reaction period (270–680 s). This stage also represents the stable decarburization period for the molten metal bath, where the added flux dissolves at a relatively slow rate in the slag. Meanwhile, the FeO produced from the gas–liquid reaction and the FeO consumed from the slag–metal reaction approach a state of dynamic equilibrium, resulting in a relatively gentle variation in the slag quantity curve during this phase. As the molten bath enters the decarburization declining period (>680 s), the oxygen decarburization efficiency decreases with the reduction of carbon content in the molten bath, leading to an increased proportion of oxygen used for oxidizing Fe and Mn. Consequently, the oxidation loss of metallic Fe increases, resulting in a rise in FeO content within the slag.

The curves depicting the changes in the compositions of the molten metal and slag, as shown in Figure 14, along with the curves representing the decarburization rate and carbon content variations during the blowing process in Figure 15, can similarly be divided into the same three stages: the oxidation of silicon and manganese into slag (or decarburization upswing period), the stable reaction period (or decarburization stable period), and the slag iron rising period (or decarburization declining period). During the rapid slag formation period (0–270 s), a significant oxidation of silicon and manganese occurs, along with some oxidation of carbon, leading to a rapid decrease in the concentrations of [Si] and [Mn] in the molten metal. The carbon content [C] initially decreases slowly before accelerating, while the decarburization rate increases with the reduction of [Si] and [Mn] concentrations in the molten metal. Once silicon and manganese have oxidized to a certain extent, the reactions within the furnace transition into the decarburization stable period (270–680 s), during which the molten metal undergoes rapid decarburization. In this phase, the carbon–oxygen reaction is primarily controlled by the mass transfer of oxygen, with the decarburization rate being influenced by the intensity of the oxygen supply. Notably, around 300–600 s during the decarburization stable period, improper on-site operations (such as failing to replenish the ore before the onset of intense decarburization and inadequately lowering the lance, resulting in insufficient FeO in the slag) led to a phenomenon known as “slag drying”. This resulted in a dip in the FeO + MnO content curve in Figure 14b and a rebound in the manganese concentration in the molten metal (referred to as “re-manganization”) in Figure 14a. Additionally, the slag drying phenomenon caused a slowdown in the actual decarburization rate, evident in the middle dip of the decarburization peak rate curve in Figure 15. As the carbon content drops below a critical threshold, the carbon–oxygen reaction shifts to being controlled by the mass transfer of carbon, marking the onset of the decarburization declining period. During this stage, the decarburization rate declines rapidly, and the reduction in oxygen consumption for decarburization means an increase in oxygen consumption for the oxidation reactions of Fe and Mn, resulting in a decrease in manganese content in the molten metal and an increase in FeO and MnO content in the slag.

In summary, the simulation results described above effectively characterize the process phenomena and variations in the outcomes during the BOF steelmaking process, providing a solid theoretical framework to explain the interconnections between relevant phenomena. By establishing a comprehensive mechanistic model, a “reproduction” of all the metallurgical reactions can be achieved in the BOF steelmaking process, thereby enhancing our understanding of related process phenomena. Furthermore, by analyzing the influence of model parameters on the metallurgical reaction outcomes, this study can offer optimization or improvement recommendations for industrial production. However, due to experimental constraints, it is challenging to obtain continuous or multiple samples of the molten metal and slag during the BOF blowing process, making it difficult to verify each of the aforementioned simulation results individually. Therefore, this study focuses on the most critical aspect of carbon content control within the blowing process, following modifications to the flue gas analysis equipment at the production site, to conduct an in-depth investigation and validation of the model simulation effectiveness.

**Figure 16:**
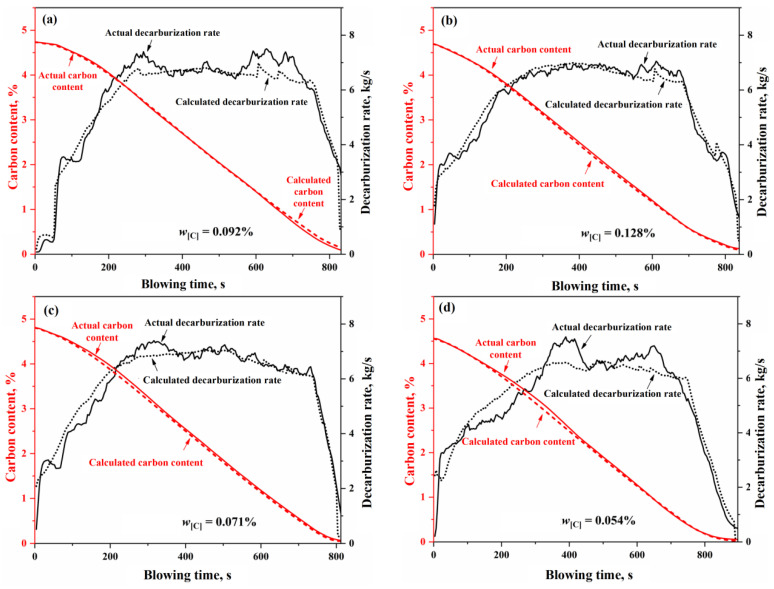
Decarburization rate and carbon content change curve of (**a**) Heat A, (**b**) Heat B, (**c**) Heat C, and (**d**) Heat D.

Based on the input material information and the sampling data at the end of the blowing process, the total decarburization during the blowing process can be calculated and compared with the total decarburization derived from flue gas analysis data (including composition and flow rate). This comparison allows for the calibration of the flue gas flow rate, ultimately resulting in continuous variation curves of the decarburization rate and carbon content based on flue gas analysis during the BOF steelmaking process. In Figure 16, the decarburization rate and carbon content curves calculated using the comprehensive multi-region reaction mechanism model for Heat A closely match the overall profile of the actual decarburization rate and carbon content curves. However, in actual BOF steelmaking processes, variations in input materials and operating conditions lead to differences in the decarburization curve profiles. Furthermore, different steel grades correspond to distinct blowing operation modes. Thus, four experimental heats were selected for simulation validation, as shown in Table 2. These heats exhibit significant differences in decarburization curve profiles and target endpoint carbon groupings, representing low-carbon ($w_{\left[C\right]}\;<\;0.06\%$), medium-carbon ($0.06\%\;\leq \;w_{\left[C\right]}\;<\;0.10\%$), and high-carbon ($w_{\left[C\right]}\;\geq \;0.10\%$) steel series. The comparison of the simulated decarburization rate and carbon content variation curves with the actual curves is illustrated in Figure 16.

In Figure 16, for the four heats with different decarburization curve profiles, the calculated decarburization rate curves exhibit a high degree of agreement with the actual decarburization rate curves, both in terms of the overall profile and in the local morphological features across the three phases of the blowing process: the decarburization increase period, the stable decarburization period, and the decarburization decline period. The relative deviations of the calculated total decarburization amounts (i.e., the cumulative values of the decarburization rates) from the actual total decarburization amounts for Heats A, B, C, and D are 1.68%, −0.45%, −0.35%, and −0.66%, respectively. Additionally, the absolute deviations between the calculated and actual carbon content variation curves are less than 0.12%, with the predicted endpoint carbon content deviations being 0.073%, −0.019%, −0.016%, and −0.028%, respectively. These results demonstrate that the comprehensive multi-region reaction mechanism model effectively characterizes the decarburization behavior during the BOF steelmaking process and provides reliable predictions for the endpoint carbon content of different target carbon groupings in steel grades.

### 5.3. Evaluation of the Comprehensive Model of the Multi-Region Reaction Mechanism

The performance of the comprehensive model of the multi-region reaction mechanism was evaluated using the coefficient of determination (R^2^), mean absolute error (MAE), and root mean square error (RMSE), as shown in Equations (8)–(10) [55]:

(8) $$R^{2}=\frac{\sum_{i=1}^{N_{p}}{\left(y_{i}^{\mathrm{act}}-\bar{y_{m}}\right)}^{2}-\sum_{i=1}^{N_{p}}{\left(y_{i}^{cal}-y_{i}^{\mathrm{act}}\right)}^{2}}{\sum_{i=1}^{N_{p}}{\left(y_{i}^{\mathrm{act}}-\bar{y_{m}}\right)}^{2}}$$

(9) $$\mathrm{MAE}=\sum_{i=1}^{N_{P}}\left|y_{i}^{cal}-y_{i}^{\mathrm{act}}\right|/N_{P}$$

(10) $$\mathrm{RMSE}=\sqrt{{\sum_{i=1}^{N_{P}}\left(y_{i}^{cal}-y_{i}^{\mathrm{act}}\right)}^{2}/N_{P}}$$

where *N_p_* is the number of data, *y*^act^ is the actual value, *y*^cal^ is the calculated value, and $\bar{y_{m}}$ is the average value.

**Figure 17:**
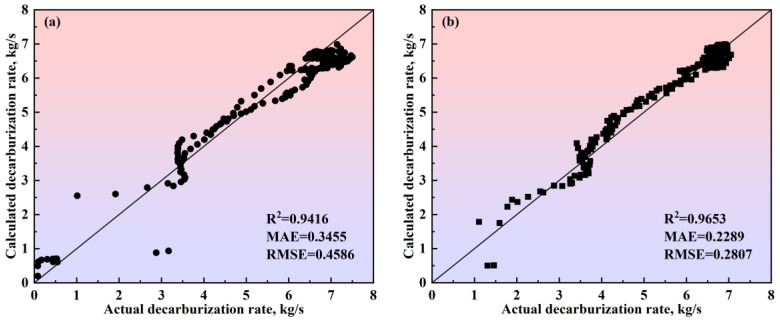

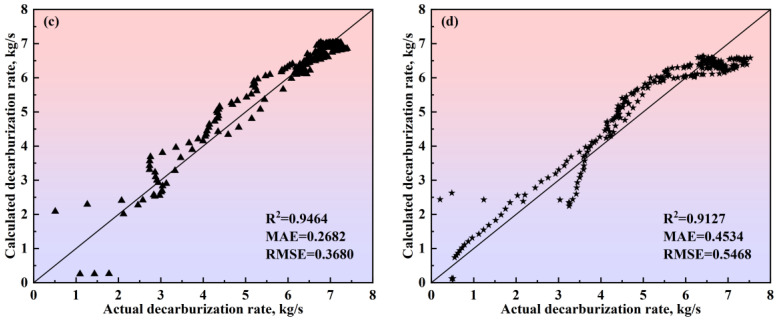
Comparison between the actual and calculated decarburization rates obtained by the comprehensive model of the multi-region reaction mechanism of (**a**) Heat A, (**b**) Heat B, (**c**) Heat C, and (**d**) Heat D. The 45-degree diagonal dotted line is the identity where the actual value is equal to the calculated value. ● represents data from Heat A; ■ represents data from Heat B; ▲ represents data from Heat C; ★ represents data from Heat D.

**Figure 18:**
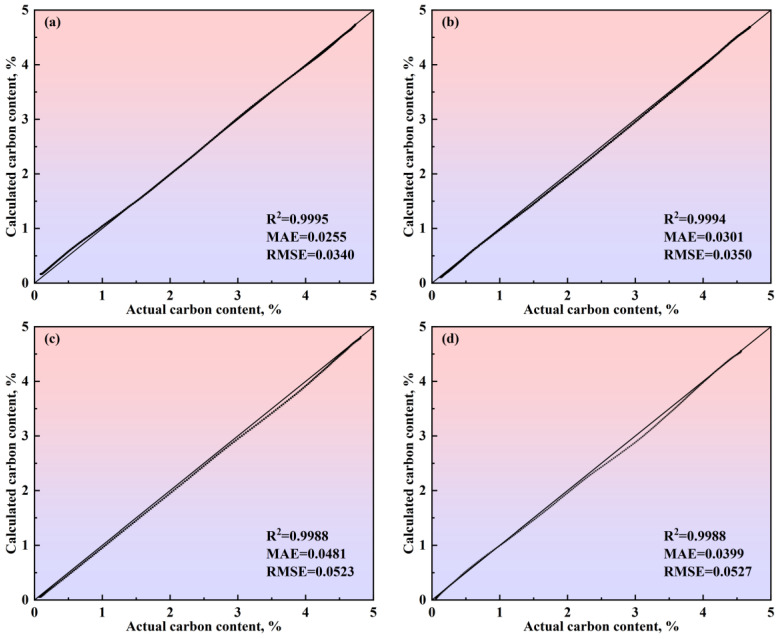
Comparison between the actual and calculated carbon content obtained by the comprehensive model of the multi-region reaction mechanism of (**a**) Heat A, (**b**) Heat B, (**c**) Heat C, and (**d**) Heat D. The 45-degree diagonal dotted line is the identity where the actual value is equal to the calculated value.

To verify the calculation accuracy of the comprehensive model of the multi-region reaction mechanism, the calculated values were compared with the actual data for different steel grades, as shown in Figure 17 and Figure 18. For the decarburization rate of Heat A, the comprehensive model’s R^2^, MAE, and RMSE are 0.9416, 0.3455, and 0.4586, respectively. For the decarburization rate of Heat B, the comprehensive model’s R^2^, MAE, and RMSE are 0.9653, 0.2289, and 0.2807, respectively. For the decarburization rate of Heat C, the comprehensive model’s R^2^, MAE, and RMSE are 0.9464, 0.2682, and 0.3680, respectively. For the decarburization rate of Heat D, the comprehensive model’s R^2^, MAE, and RMSE are 0.9127, 0.4534, and 0.5468, respectively. For the carbon content of Heat A, the comprehensive model’s R^2^, MAE, and RMSE are 0.9995, 0.0255, and 0.0340, respectively. For the carbon content of Heat B, the comprehensive model’s R^2^, MAE, and RMSE are 0.9994, 0.0301, and 0.0350, respectively. For the carbon content of Heat C, the comprehensive model’s R^2^, MAE, and RMSE are 0.9988, 0.0481, and 0.0523, respectively. For the carbon content of Heat D, the comprehensive model’s R^2^, MAE, and RMSE are 0.9988, 0.0399, and 0.0527, respectively. In addition, the closer the scatter to the 45-degree diagonal line, the smaller the error between the calculated and actual values. Meanwhile, this figure shows that, for the decarburization rate and carbon content of Heat A, Heat B, Heat C, and Heat D, the scatter points are distributed around the 45-degree diagonal line, indicating the comprehensive model of the multi-region reaction mechanism’s high calculation accuracy.

In this study, a comprehensive mechanism model was established for simulating the BOF steelmaking process based on an in-depth analysis of steelmaking process behaviors. This model can realize the dynamic “reproduction” of metallurgical reactions throughout the entire BOF steelmaking process, thereby enhancing our understanding of various process behaviors and phenomena while also validating the model’s effectiveness and universality. Furthermore, this study can provide a reference for parameter optimization of the industrial model by analyzing the influence of relevant model parameters on the BOF blowing process. With the rapid development of artificial intelligence (AI) technology, it can extract complex nonlinear relationships in industrial modeling, identify potential patterns or rules in data, and intelligently optimize models based on actual data. By automatically analyzing and learning from a large amount of historical data, AI can discover complex correlations that are difficult for traditional methods to capture, achieving more accurate predictions and optimizations. This enables industrial processes to operate more precisely and efficiently while reducing human intervention and reliance on experience and enhancing the adaptability and decision-making capabilities of the models. In the future, we will introduce AI algorithms on the basis of this research to further enhance the accuracy and reliability of the comprehensive model, laying a foundation for the intelligentization of steelmaking.

## 6. Conclusions

In this paper, the reaction mechanisms of six reaction zones within the BOF were analyzed. Subsequently, a comprehensive mechanism model for multi-zone reactions in the BOF was established. Finally, FactSage thermodynamic calculation software was employed to simulate the blowing process in the BOF. The main research conclusions are as follows:

1. The oxygen distribution ratio among the elements in the gas–liquid reactions of the jet impact zone is determined by the Gibbs free energy contributions of each reaction. The kinetic conditions for the slag–metal reactions in the molten bath surface zone are primarily controlled by bottom blowing, which contributes approximately 80% to the overall stirring energy. Under typical operating conditions, the effective mass transfer coefficient on the molten metal side is (3.08–23.18) × 10^−4^ m/s, while, on the slag side, it is (12.42–14.38) × 10^−5^ m/s. Additionally, the overall effective decarburization rate of the droplets ranges from 0.47 to 0.87.
2. In the three auxiliary reaction zones, approximately 6% of the top-blown oxygen is utilized for the secondary combustion of the furnace gas in the gas phase homogenization zone. The melting of scrap steel is influenced by both the carbon concentration and thermal drives, while the melting of pig iron is primarily controlled by thermal mechanisms. The dissolution rate of CaO and MgO fluxes in the slag homogenization zone is determined by the concentration gradient and the stirring intensity of the BOF. The dissolution rates of the fluxes are relatively fast in the early and late stages of the blowing process, while they are slower during the mid-process.
3. The multiphase reaction zone was divided into three main reaction zones and three auxiliary reaction zones in the converter, and the reaction mechanisms of the six zones were analyzed from both thermodynamic and kinetic perspectives. Based on this, a multi-zone reaction mechanism model was established, and the accuracy of the model was verified according to actual production data. The results show that the calculated decarburization rate curves and carbon content curves derived from this comprehensive model closely match both the overall profiles and local morphological features of the actual curves from the furnace operation. The relative deviation of the cumulative decarburization rate (i.e., total decarburization amount) ranges from −0.66% to 1.68%, while the absolute deviation of the calculated carbon content curve compared to the actual curve is less than 0.12%. The prediction error for the final carbon content is −0.028–0.073%.

## Figures and Tables

**Table 1:** Relevant parameters of Heat A.

Name	Value	Name	Value	Name	Value
Weight of molten iron, t	108.0	[C] of molten iron, %	4.73	[C] of molten metal at the end of blowing process, %	0.092
Weight of scrap steel, t	16.2	[Mn] of molten iron, %	0.36	[Mn] of molten metal at the end of blowing process, %	0.163
Weight of lime, kg	3524	[Si] of molten iron, %	0.50	[Si] of molten metal at the end of blowing process, %	0.002
Weight of dolomite, kg	1028	Temperature of molten iron, °C	1325	Temperature of molten metal at the end of blowing process, °C	1640
Weight of ore, kg	622	Total amount of oxygen blown, Nm^3^	5316	Bottom blowing gas intensity, Nm^3^/(t·min)	0.03~0.07

**Table 2:** Information on the experimental heats.

Heat	Grade of Steel	Target [C] of Molten Metal at the End of Blowing Process	Calculated [C] of Molten Metal at the End of Blowing Process, %	Actual [C] of Molten Metal at the End of Blowing Process, %	Remark
A	B/Q345B	$w_{\left[C\right]}\;\geq \;0.10\%$	0.165	0.092	Abnormal blowing, the slag becomes dry
B	65Mn	$w_{\left[C\right]}\;\geq \;0.10\%$	0.109	0.128	Normal blowing
C	510L	$0.06\%\;\leq \;w_{\left[C\right]}\;<\;0.10\%$	0.055	0.071	Normal blowing
D	200IF	$w_{\left[C\right]}\;<\;0.06\%$	0.026	0.054	Normal blowing

## Data Availability

No new data were created or analyzed in this study.

## Supplementary Materials

The following supporting information can be downloaded at https://www.mdpi.com/article/10.3390/ma18051038/s1: Section S1.1: Direct oxidation reaction of different elements in molten metal; Section S1.2: Calculation of oxygen distribution ratio of each element; Section S1.3: Composition calculation of molten metal and slag; Section S2.1: Variation of each component in molten steel and slag; Section S2.2: Calculation of circulation renewal rate at slag-metal interface; Section S2.3: Calculation of mass transfer coefficient of slag and gold and combined blowing stirring energy; Section S3.1: Formula derivation of decarburization rate for metal droplet; Section S3.2: Calculation of droplet generation and its particle size distribution; Section S3.3: Relationship between effective decarburization rate of droplet and initial diameter; Section S4: Oxygen ratio calculation for direct oxidation reaction and secondary combustion reaction of furnace gas in impact zone; Section S5: Melting reaction of scrap in metal mixing zone; Section S6: Dissolution reaction of flux in the mixed slag zone.
