# Supplementary material for: Analysis of Multi-Zone Reaction Mechanisms in BOF Steelmaking and Comprehensive Simulation
Source: Materials (Basel). 2025 Feb 26;18(5):1038. doi: 10.3390/ma18051038 (PMC11901173; doi:10.3390/ma18051038)
Supplement: Supplementary file 1 [file materials-18-01038-s001.zip › materials-3473235-supplementary.pdf]

Supplementary Information

S1 Gas-liquid reaction in the jet impact zone

S1.1 Direct oxidation reaction of different elements in molten metal

The direct oxidation reactions of elements such as C, Si, Mn, P, and Fe in the molten metal are represented by the following thermodynamic equations [1], as shown in **Equations (S1) to (S5)**.

$$[\mathrm{C}] + \frac{1}{2}\mathrm{O_2} = \mathrm{CO}\ ,\quad \Delta G_{\mathrm{C}}^{\Theta} = -136900 - 43.51T \quad (\mathrm{J/mol}) \tag{S1}$$

$$[\mathrm{Si}] + \mathrm{O_2} = (\mathrm{SiO_2})\ ,\quad \Delta G_{\mathrm{Si}}^{\Theta} = -817051 + 217.27T \quad (\mathrm{J/mol}) \tag{S2}$$

$$[\mathrm{Mn}] + \frac{1}{2}\mathrm{O_2} = (\mathrm{MnO}),\quad \Delta G_{\mathrm{Mn}}^{\Theta} = -361495 + 111.63T \quad (\mathrm{J/mol}) \tag{S3}$$

$$[\mathrm{P}] + \frac{5}{4}\mathrm{O_2} = \frac{1}{2}(\mathrm{P_2O_5})\ ,\quad \Delta G_{\mathrm{P}}^{\Theta} = -645049 + 270.30T \quad (\mathrm{J/mol}) \tag{S4}$$

$$[\mathrm{Fe}] + \frac{1}{2}\mathrm{O_2} = (\mathrm{FeO})\ ,\quad \Delta G_{\mathrm{Fe}}^{\Theta} = -237956 + 49.43T \quad (\mathrm{J/mol}) \tag{S5}$$

Based on Equations (S1) to (S5), the standard Gibbs free energy changes for the oxidation reactions of C, Si, Mn, and Fe were calculated.

S1.2 Calculation of oxygen distribution ratio of each element

S1.2.1 Calculation of oxygen distribution ratios for each element under standard conditions

The relationship between the oxygen distribution ratios of each element's reaction and Gibbs free energy is illustrated in **Equations (S6) to (S11)**.

$$\sum_i \Delta G_i = \Delta G_{\mathrm{C}} + \frac{1}{2}\Delta G_{\mathrm{Si}} + \Delta G_{\mathrm{Mn}} + \Delta G_{\mathrm{Fe}} \tag{S6}$$

$$x_{\mathrm{O_2}}^{\mathrm{C}} = \frac{\Delta G_{\mathrm{C}}}{\sum_i \Delta G_i} \tag{S7}$$

$$x_{\mathrm{O_2}}^{\mathrm{Si}} = \frac{1}{2}\cdot\frac{\Delta G_{\mathrm{Si}}}{\sum_i \Delta G_i} \tag{S8}$$

$$x_{\mathrm{O_2}}^{\mathrm{Mn}} = \frac{\Delta G_{\mathrm{Mn}}}{\sum_i \Delta G_i} \tag{S9}$$

$$x_{\mathrm{O_2}}^{\mathrm{Fe}} = \frac{\Delta G_{\mathrm{Fe}}}{\sum_i \Delta G_i} \tag{S10}$$

$$\sum_i x_{\mathrm{O_2}}^{i} = x_{\mathrm{O_2}}^{\mathrm{C}} + x_{\mathrm{O_2}}^{\mathrm{Si}} + x_{\mathrm{O_2}}^{\mathrm{Mn}} + x_{\mathrm{O_2}}^{\mathrm{Fe}} = 1 \tag{S11}$$

Based on Equations (S1) to (S5), the standard Gibbs free energy changes for direct oxidation reactions can be obtained, and by substituting these values into Equations (S6) to (S11), the oxygen distribution ratios under standard conditions can be determined.

S1.2.2 Calculation of oxygen distribution ratio of each element under actual production conditions

Under actual production conditions, the Gibbs free energy changes for the oxidation reactions of C, Si, Mn, and Fe are obtained based on the van der Hoff isothermal equations, as shown in **Equations (S12) and (S13)**.

$$\Delta G_{\mathrm{C}} = \Delta G_{\mathrm{C}}^{\Theta} + RT\ln\left[\frac{\left(P_{\mathrm{CO}}/P^{\Theta}\right)}{a_{[\mathrm{C}]}\cdot\left(P_{\mathrm{O_2}}/P^{\Theta}\right)^{1/2}}\right] \tag{S12}$$

$$\Delta G_{i} = \Delta G_{i}^{\Theta} + RT\ln\left[\frac{1}{a_{[i]}\cdot\left(P_{\mathrm{O_2}}/P^{\Theta}\right)}\right] \quad , \left(i = \mathrm{Si、Mn、Fe}\right) \tag{S13}$$

By substituting actual parameters such as temperature and component activities into Equations (S12) and (S13), the ΔG for each chemical reaction can be calculated. Subsequently, the oxygen distribution ratio in the gas-liquid reactions under actual production conditions can be obtained based on Equations (S6) to (S11).

S1.3 Composition calculation of molten metal and slag

Assuming that the amounts of molten metal and oxygen participating in the gas-liquid reactions in the impact zone per unit time are represented by $W_{\mathrm{metal}}^{\mathrm{IZ}}$ and $W_{\mathrm{O_2}}^{\mathrm{IZ}}$, respectively, the mass of each element in the molten metal varies with time as shown in **Equations (S14) to (S17)**.

$$\frac{\mathrm{d}\left(W_{\mathrm{metal}}^{\mathrm{IZ}}C_{[\mathrm{C}]}^{\mathrm{b}}\right)}{\mathrm{d}t} = W_{\mathrm{O_2}}^{\mathrm{IZ}}\cdot\left(\frac{12}{16}\cdot x_{\mathrm{O}}^{\mathrm{C}}\right) \tag{S14}$$

$$\frac{\mathrm{d}\left(W_{\mathrm{metal}}^{\mathrm{IZ}}C_{[\mathrm{Si}]}^{\mathrm{b}}\right)}{\mathrm{d}t} = W_{\mathrm{O_2}}^{\mathrm{IZ}}\cdot\left(\frac{28}{32}\cdot x_{\mathrm{O}}^{\mathrm{Si}}\right) \tag{S15}$$

$$\frac{\mathrm{d}\left(W_{\mathrm{metal}}^{\mathrm{IZ}}C_{[\mathrm{Mn}]}^{\mathrm{b}}\right)}{\mathrm{d}t} = W_{\mathrm{O_2}}^{\mathrm{IZ}}\cdot\left(\frac{55}{16}\cdot x_{\mathrm{O}}^{\mathrm{Mn}}\right) \tag{S16}$$

$$\frac{\mathrm{d}\left(W_{[\mathrm{Fe}]}\right)}{\mathrm{d}t} = W_{\mathrm{O_2}}^{\mathrm{IZ}}\cdot\left(\frac{56}{16}\cdot x_{\mathrm{O}}^{\mathrm{Fe}}\right) \tag{S17}$$

In the oxidation products of the gas-liquid reactions, CO and CO_2 enter the flue gas, while other products are transferred to the slag. The variation in the amounts of each product over time is shown in **Equations (S18) to (S22)**.

$$\frac{\mathrm{d}W_{\mathrm{CO}}}{\mathrm{d}t} = W_{\mathrm{O_2}}^{\mathrm{IZ}}\cdot\left(\frac{12}{16}\cdot x_{\mathrm{O}}^{\mathrm{C}}\right)\cdot\left(1-PCR\right)\cdot M_{\mathrm{CO}} \tag{S18}$$

$$\frac{\mathrm{d}W_{\mathrm{CO_2}}}{\mathrm{d}t} = W_{\mathrm{O_2}}^{\mathrm{IZ}}\cdot\left(\frac{12}{16}\cdot x_{\mathrm{O}}^{\mathrm{C}}\right)\cdot PCR\cdot M_{\mathrm{CO_2}} \tag{S19}$$

$$\frac{\mathrm{d}W_{(\mathrm{SiO_2})}}{\mathrm{d}t} = W_{\mathrm{O_2}}^{\mathrm{IZ}}\cdot\left(\frac{28}{32}\cdot x_{\mathrm{O}}^{\mathrm{Si}}\right)\cdot M_{\mathrm{SiO_2}} \tag{S20}$$

$$\frac{\mathrm{d}W_{(\mathrm{MnO})}}{\mathrm{d}t} = W_{\mathrm{O_2}}^{\mathrm{IZ}}\cdot\left(\frac{55}{16}\cdot x_{\mathrm{O}}^{\mathrm{Mn}}\right)\cdot M_{\mathrm{MnO}} \tag{S21}$$

$$\frac{\mathrm{d}W_{(\mathrm{FeO})}}{\mathrm{d}t} = W_{\mathrm{O}_2}^{\mathrm{IZ}} \cdot \left(\frac{56}{16} \cdot x_{\mathrm{O}}^{\mathrm{Fe}}\right) \cdot M_{\mathrm{FeO}} \tag{S22}$$

Based on the above equations, the reaction quantities of each chemical reaction in the oxygen jet reaction zone can be calculated, along with their effects on the composition changes of the molten metal and slag.

S2 Slag-metal reaction in molten bath surface zone

S2.1 Variation of each component in molten steel and slag

The equations for the indirect oxidation reactions of various elements at the slag-metal interface are shown in **Equations (S23) to (S27)**.

$$(\mathrm{FeO}) = [\mathrm{O}] + [\mathrm{Fe}] \tag{S23}$$

$$[\mathrm{C}] + (\mathrm{FeO}) = \mathrm{CO}_{(g)} + [\mathrm{Fe}] \tag{S24}$$

$$[\mathrm{Si}] + 2(\mathrm{FeO}) = (\mathrm{SiO_2}) + 2[\mathrm{Fe}] \tag{S25}$$

$$[\mathrm{Mn}] + (\mathrm{FeO}) = (\mathrm{MnO}) + [\mathrm{Fe}] \tag{S26}$$

$$2[\mathrm{P}] + 5(\mathrm{FeO}) = (\mathrm{P_2O_5}) + 5[\mathrm{Fe}] \tag{S27}$$

The mass transfer rates of the reactants in the molten metal side and the products in the molten slag side are represented in **Equations (S28) and (S29)**, respectively.

$$R_i = A_{\mathrm{sm}} k_i \left(C_i^{\mathrm{b}} - C_i^*\right) = A_{\mathrm{sm}} k_i \rho_m \frac{\left(w_i^{\mathrm{b}} - w_i^*\right)}{100 \cdot M_i} \tag{S28}$$

$$R_j = A_{\mathrm{sm}} k_j \left(C_j^{\mathrm{b}} - C_j^*\right) = A_{\mathrm{sm}} k_j \rho_{\mathrm{s}} \frac{\left(w_j^{\mathrm{b}} - w_j^*\right)}{100 \cdot M_j} \tag{S29}$$

The thermodynamic equilibrium constants for the chemical reactions illustrated in Equations (S23) to (S27) are presented in **Equations (S30) and (S34).**

$$K_{\mathrm{FeO}} = \frac{a_{[\mathrm{Fe}]} a_{[\mathrm{O}]}}{a_{(\mathrm{FeO})}} = \frac{f_{[\mathrm{O}]} w_{[\mathrm{O}]}^*}{\gamma_{(\mathrm{FeO})} X_{(\mathrm{FeO})}^*} \tag{S30}$$

$$K_{\mathrm{C}} = \frac{a_{[\mathrm{Fe}]}\, P_{\mathrm{CO}}/P^{\Theta}}{a_{(\mathrm{FeO})} a_{[\mathrm{C}]}} = \frac{P_{\mathrm{CO}}/P^{\Theta}}{\gamma_{(\mathrm{FeO})} X_{(\mathrm{FeO})}^* f_{[\mathrm{C}]} w_{[\mathrm{C}]}^*} \tag{S31}$$

$$K_{\mathrm{Si}} = \frac{a_{[\mathrm{Fe}]} a_{(\mathrm{SiO_2})}}{a_{(\mathrm{FeO})} a_{[\mathrm{Si}]}} = \frac{\gamma_{(\mathrm{SiO_2})} X_{(\mathrm{SiO_2})}^*}{\gamma_{(\mathrm{FeO})} X_{(\mathrm{FeO})}^* f_{[\mathrm{Si}]} w_{[\mathrm{Si}]}^*} \tag{S32}$$

$$K_{\mathrm{Mn}} = \frac{a_{[\mathrm{Fe}]} a_{(\mathrm{MnO})}}{a_{(\mathrm{FeO})} a_{[\mathrm{Mn}]}} = \frac{\gamma_{(\mathrm{MnO})} X_{(\mathrm{MnO})}^*}{\gamma_{(\mathrm{FeO})} X_{(\mathrm{FeO})}^* f_{[\mathrm{Mn}]} w_{[\mathrm{Mn}]}^*} \tag{S33}$$

$$K_{\mathrm{P}} = \frac{a_{[\mathrm{Fe}]}^5 a_{(\mathrm{P_2O_5})}}{a_{[\mathrm{P}]}^2 a_{(\mathrm{FeO})}^5} = \frac{\gamma_{(\mathrm{P_2O_5})} X_{(\mathrm{P_2O_5})}^*}{\left[\gamma_{(\mathrm{FeO})} X_{(\mathrm{FeO})}^*\right]^5 \left(f_{[\mathrm{P}]} w_{[\mathrm{P}]}^*\right)^2} \tag{S34}$$

The activity of the components in the aforementioned equations is calculated based on the composition of the slag and molten metal. In the case of the molten metal, the activity of the dissolved elements is derived from Henry's law activity, utilizing a standard

state of 1% mass concentration. As an illustration, the calculation formula for the activity coefficient of carbon is presented in **Equation (S35)**.

$$\lg f_{\mathrm{C}} = \sum e_{\mathrm{C}}^{i} w_{[i]} \tag{S35}$$

The activities of other elements in the molten metal can be calculated using the same methodology, and the interaction coefficients for each element are derived from the thermodynamic data handbook for steelmaking [2], as presented in **Table S1**.

Table S1. Interaction coefficients of elements in molten metal.

i	C	Si	Mn	P	O
e_{C}^{i}	0.243	$162/T-0.008$	-0.0084	0.051	-0.32
e_{Si}^{i}	0.18	-0.103	-0.0146	0.09	-0.119
e_{Mn}^{i}	$-1370/T+0.690$	$-1838/T+0.964$	0	-0.06	-0.083
e_{P}^{i}	0.126	0.099	-0.032	0.054	0.13
e_{O}^{i}	-0.421	-0.066	-0.021	0.07	$-1750/T+0.76$

The activity of each component within the slag is defined by **Equation (S36)**.

$$a_j = \gamma_j \cdot X_j \tag{S36}$$

The calculations of the activity coefficients for the components within the slag are detailed in **Equations (S37) to (S40)**[2].

$$\left\{ \begin{array}{ll} T\ln\gamma_{\mathrm{SiO_2}} = -1176.7 - 7427.6\times\dfrac{X_{\mathrm{FeO}}}{X_{\mathrm{SiO_2}}} + 1662.2\times\left(\dfrac{X_{\mathrm{FeO}}}{X_{\mathrm{SiO_2}}}\right)^2 & , \dfrac{X_{\mathrm{FeO}}}{X_{\mathrm{SiO_2}}} < 1 \\ \gamma_{\mathrm{SiO_2}} = 0.005 & , \dfrac{X_{\mathrm{FeO}}}{X_{\mathrm{SiO_2}}} \geq 1 \end{array} \right\} \tag{S37}$$

$$\left\{ \begin{array}{ll} \gamma_{\mathrm{MnO}} = -0.07\times B^3 + 1.012\times B^2 - 5.026\times B + 9.853 & , B > 2.239 \\ \gamma_{\mathrm{MnO}} = 0.569\times B^3 - 1.176\times B^2 + 0.979\times B + 0.123 & , B \leq 2.239 \end{array} \right\} \tag{S38}$$

$$\log\gamma_{\mathrm{FeO}} = \frac{1262}{T} - 1.1302X_{\mathrm{FeO}} + 0.96X_{\mathrm{SiO_2}} + 0.123X_{\mathrm{CaO}} - 0.4198 \tag{S39}$$

$$\log\gamma_{\mathrm{P_2O_5}} = \frac{1007}{T} - 4.995X_{\mathrm{FeO}} + 1.377X_{\mathrm{SiO_2}} - 6.775X_{\mathrm{CaO}} + 2.816X_{\mathrm{MgO}} - 13.992 \tag{S40}$$

For the thermodynamic equilibrium reactions at the slag-metal interface, the mass transfer rates of reactants and products adhere to the relationships defined in **Equations (S41) to (S44)**.

$$R_{[\mathrm{Si}]} = R_{(\mathrm{SiO_2})} \tag{S41}$$

$$R_{[\mathrm{Mn}]} = R_{(\mathrm{MnO})} \tag{S42}$$

$$2R_{[\mathrm{P}]} = R_{(\mathrm{P_2O_5})} \tag{S43}$$

$$-R_{(\mathrm{FeO})} = R_{[\mathrm{C}]} + \frac{1}{2}R_{[\mathrm{Si}]} + R_{[\mathrm{Mn}]} + \frac{5}{2}R_{[\mathrm{P}]} - R_{[\mathrm{O}]} \tag{S44}$$

By solving the system of Equations (S28) to (S44) through an iterative approach, the equilibrium concentrations of each component (w_i^*, w_j^*) at the slag-metal reaction inter-

face are determined. Subsequently, the changes in the compositions of steel and slag induced by the reactions at the slag-metal interface can be calculated, with the calculation formulas provided in **Equations (S45) to (S53)**.

$$\frac{\mathrm{d}\left(W_{\mathrm{metal}}^{\mathrm{BZ}} w_{[\mathrm{O}]}^{\mathrm{b}}\right)}{\mathrm{d}t} = -A_{\mathrm{sm}} k_{\mathrm{O}} \rho_{\mathrm{m}} \left(w_{[\mathrm{O}]}^{\mathrm{b}} - w_{[\mathrm{O}]}^{*}\right) \tag{S45}$$

$$\frac{\mathrm{d}\left(W_{\mathrm{metal}}^{\mathrm{BZ}} w_{[\mathrm{C}]}^{\mathrm{b}}\right)}{\mathrm{d}t} = -A_{\mathrm{sm}} k_{\mathrm{C}} \rho_{\mathrm{m}} \left(w_{[\mathrm{C}]}^{\mathrm{b}} - w_{[\mathrm{C}]}^{*}\right) \tag{S46}$$

$$\frac{\mathrm{d}\left(W_{\mathrm{metal}}^{\mathrm{BZ}} w_{[\mathrm{Si}]}^{\mathrm{b}}\right)}{\mathrm{d}t} = -A_{\mathrm{sm}} k_{\mathrm{Si}} \rho_{\mathrm{m}} \left(w_{[\mathrm{Si}]}^{\mathrm{b}} - w_{[\mathrm{Si}]}^{*}\right) \tag{S47}$$

$$\frac{\mathrm{d}\left(W_{\mathrm{metal}}^{\mathrm{BZ}} w_{[\mathrm{Mn}]}^{\mathrm{b}}\right)}{\mathrm{d}t} = -A_{\mathrm{sm}} k_{\mathrm{Mn}} \rho_{\mathrm{m}} \left(w_{[\mathrm{Mn}]}^{\mathrm{b}} - w_{[\mathrm{Mn}]}^{*}\right) \tag{S48}$$

$$\frac{\mathrm{d}\left(W_{\mathrm{metal}}^{\mathrm{BZ}} w_{[\mathrm{P}]}^{\mathrm{b}}\right)}{\mathrm{d}t} = -A_{\mathrm{sm}} k_{\mathrm{P}} \rho_{\mathrm{m}} \left(w_{[\mathrm{P}]}^{\mathrm{b}} - w_{[\mathrm{P}]}^{*}\right) \tag{S49}$$

$$\frac{\mathrm{d}\left(W_{\mathrm{SiO_2}}\right)}{\mathrm{d}t} = A_{\mathrm{sm}} k_{\mathrm{Si}} \rho_{\mathrm{m}} \left(w_{[\mathrm{Si}]}^{\mathrm{b}} - w_{[\mathrm{Si}]}^{*}\right) M_{\mathrm{SiO_2}} / M_{\mathrm{Si}} \tag{S50}$$

$$\frac{\mathrm{d}\left(W_{\mathrm{MnO}}\right)}{\mathrm{d}t} = A_{\mathrm{sm}} k_{\mathrm{Mn}} \rho_{\mathrm{m}} \left(w_{[\mathrm{Mn}]}^{\mathrm{b}} - w_{[\mathrm{Mn}]}^{*}\right) M_{\mathrm{MnO}} / M_{\mathrm{Mn}} \tag{S51}$$

$$\frac{\mathrm{d}\left(W_{\mathrm{P_2O_5}}\right)}{\mathrm{d}t} = \frac{1}{2} A_{\mathrm{sm}} k_{\mathrm{P}} \rho_{\mathrm{m}} \left(w_{[\mathrm{P}]}^{\mathrm{b}} - w_{[\mathrm{P}]}^{*}\right) M_{\mathrm{P_2O_5}} / M_{\mathrm{P}} \tag{S52}$$

$$\frac{\mathrm{d}\left(W_{\mathrm{FeO}}\right)}{\mathrm{d}t} = \left\{ \begin{array}{l} \dfrac{\mathrm{d}\left(W_{\mathrm{metal}}^{\mathrm{BZ}} w_{[\mathrm{O}]}^{\mathrm{b}}\right)}{\mathrm{d}t} \cdot \dfrac{M_{\mathrm{FeO}}}{M_{\mathrm{O}}} + \dfrac{\mathrm{d}\left(W_{\mathrm{metal}}^{\mathrm{BZ}} w_{[\mathrm{C}]}^{\mathrm{b}}\right)}{\mathrm{d}t} \cdot \dfrac{M_{\mathrm{FeO}}}{M_{\mathrm{C}}} + \dfrac{1}{2} \cdot \dfrac{\mathrm{d}\left(W_{\mathrm{metal}}^{\mathrm{BZ}} w_{[\mathrm{Si}]}^{\mathrm{b}}\right)}{\mathrm{d}t} \cdot \dfrac{M_{\mathrm{FeO}}}{M_{\mathrm{Si}}} \\ + \dfrac{\mathrm{d}\left(W_{\mathrm{metal}}^{\mathrm{BZ}} w_{[\mathrm{Mn}]}^{\mathrm{b}}\right)}{\mathrm{d}t} \cdot \dfrac{M_{\mathrm{FeO}}}{M_{\mathrm{Mn}}} + \dfrac{5}{2} \cdot \dfrac{\mathrm{d}\left(W_{\mathrm{metal}}^{\mathrm{BZ}} w_{[\mathrm{P}]}^{\mathrm{b}}\right)}{\mathrm{d}t} \dfrac{M_{\mathrm{FeO}}}{M_{\mathrm{P}}} \end{array} \right\} \tag{S53}$$

S2.2 Calculation of circulation renewal rate at slag-metal interface

The circulation renewal rate at the slag-metal interface (u_{sm}) is the sum of the effects from both top-blowing and bottom-blowing, as shown in **Equation (S54)**.

$$u_{\mathrm{sm}} = u_l + u_{\mathrm{bottom}} \tag{S54}$$

The flow rate under top-blowing conditions is influenced by the morphology of the impact crater. The formulas for calculating flow rate in both conventional and high lance position modes are provided in **Equations (S55) and (S56)**, respectively [3].

$$100 \times u_l \times \cos\theta = \left(0.026 \pm 0.004\right) \times \sqrt{100 \times h_{\mathrm{cav}}} - \left(0.020 \pm 0.006\right) \tag{S55}$$

$$100 \times u_l \times \cos\theta = \left(0.018 \pm 0.002\right) \times \sqrt{100 \times h_{\mathrm{cav}}} - \left(0.014 \pm 0.003\right) \tag{S56}$$

The circulation renewal rate under bottom-blowing conditions (u_{bottom}) is determined by calculations outlined in **Equations (S57) to (S59)** [4].

$$u_{\mathrm{bottom}} = 1.16 \times \left(Q_{\mathrm{B}}^{*}\right)^{0.32} \left(z^{*}\right)^{-0.28} \times \sqrt{gH_{\mathrm{bath}}} \tag{S57}$$

$$Q_{\mathrm{B}}^{*}=\frac{Q_{\mathrm{B}}}{g^{0.5}H_{\mathrm{bath}}^{2.5}} \tag{S58}$$

$$z_{\mathrm{m}}^{*}=\frac{z_{\mathrm{m}}}{H_{\mathrm{bath}}} \tag{S59}$$

At the surface of the molten bath, where $z_{\mathrm{m}}=H_{\mathrm{bath}}$ at the slag-metal interface, substitute into Equations (S57) to (S59) and arrange to obtain **Equation (S60)**.

$$u_{\mathrm{bottom}}=1.16\times\frac{Q_{\mathrm{B}}^{0.32}g^{0.34}}{H_{\mathrm{bath}}^{0.3}} \tag{S60}$$

The values of u_l and u_{bottom} under the typical conditions of this study can be calculated based on Equations (S55) to (S60).

S2.3 Calculation of mass transfer coefficient of slag and gold and combined blowing stirring energy

Utilizing the effective equilibrium reaction theory and the reaction volume method, it is assumed that the mass transfer rate of the components in the effective equilibrium reaction zone is the same [5]. The mass transfer coefficients for the metal (k_{m}) and slag (k_{s}) sides within this zone are calculated using **Equations (S61) and (S62)**, respectively [6][7].

$$\log k_{\mathrm{m}}=1.98+0.5\cdot\log\left(\frac{\varepsilon_{\mathrm{tot}}H_{\mathrm{bath}}{}^{2}}{100D_{\mathrm{vessel}}}\right)-\frac{125000}{2.3\left(R\cdot T_{\mathrm{m}}\right)} \tag{S61}$$

$$k_{\mathrm{s}}=0.5507\times\exp\left\{0.7124\times\log\left[\frac{\varepsilon_{\mathrm{B}}}{\rho_{\mathrm{m}}}\times\left(\frac{D_{\mathrm{vessel}}^{2}}{H_{\mathrm{bath}}}\right)\right]\right\} \tag{S62}$$

$$\varepsilon_{\mathrm{B}}=6.18\cdot\frac{Q_{\mathrm{B}}T_{\mathrm{m}}}{V_{\mathrm{m}}}\cdot\left[2.3\cdot\log\left(1+\frac{\rho_{\mathrm{m}}gH_{\mathrm{bath}}}{P_{\mathrm{a}}}\right)+\eta\cdot\left(1-\frac{T_{\mathrm{g}}}{T_{\mathrm{m}}}\right)\right] \tag{S63}$$

$$\varepsilon_{\mathrm{T}}=6.32\times10^{-7}\cdot\frac{\cos\alpha}{V_{\mathrm{m}}}\cdot\frac{Q_{\mathrm{T}}{}^{3}M_{\mathrm{O_2}}}{N^{2}d_{\mathrm{e}}{}^{3}x} \tag{S64}$$

$$\varepsilon_{\mathrm{tot}}=\varepsilon_{\mathrm{B}}+\lambda\varepsilon_{\mathrm{T}} \tag{S65}$$

Utilizing Equations (S63) to (S65), the comprehensive stirring energy for the combined blowing process of the converter under the typical working conditions of this study can be calculated. Additionally, according to Equations (S61) and (S62), the variations in the effective mass transfer rates of the slag and metal phases in the molten bath surface zone during the blowing process under the typical working conditions of this study can be calculated.

S3 Metal droplet reaction in emulsion zone

S3.1 Formula derivation of decarburization rate for metal droplet

In our previous research [8], we derived the **Equation (S66)** for the decarbonization rate of metal droplets.

$$\frac{\mathrm{d}w_{[\mathrm{C}]}}{\mathrm{d}t}=\begin{cases}\frac{1}{50}\cdot\sqrt{\frac{D_{\mathrm{FeO}}u_d}{\pi d^3}}\cdot\left(1-\frac{f_{\mathrm{solid}}}{f^*_{\mathrm{solid}}}\right)^{1.25}\cdot\left(1-f_{\mathrm{solid}}\right)^{2/3}\cdot\frac{\rho_s}{\rho_d}\cdot\left[w^{\mathrm{b}}_{(\mathrm{FeO})}-w^*_{(\mathrm{FeO})}\right] & , \left(J_{\mathrm{FeO}}/J_C\right)\leqslant 1\\ \frac{3}{25}\cdot\sqrt{\frac{D_{\mathrm{C}}u_d}{\pi d^3}}\cdot\left(1-f_{\mathrm{solid}}\right)^{2/3}\cdot\left(w^{\mathrm{b}}_{[\mathrm{C}]}-w^*_{[\mathrm{C}]}\right) & , \left(J_{\mathrm{FeO}}/J_C\right)>1\end{cases}\tag{S66}$$

The parameters $w^*_{[\mathrm{C}]}$ and $w^*_{(\mathrm{FeO})}$ in Equation (S64) are determined by the corresponding chemical reaction **Equation (S67)**.

$$[\mathrm{C}]+(\mathrm{FeO})=\mathrm{CO}+[\mathrm{Fe}]\qquad \log K^{\Theta}=-\frac{5160}{T}+4.74\tag{S67}$$

The chemical equilibrium constant (K^{Θ}) for the reaction in Equation (S67) is presented in **Equation (S68)**.

$$K^{\Theta}=\frac{P_{\mathrm{CO}}}{a_{\mathrm{C}}\cdot a_{(\mathrm{FeO})}}=\frac{P_{\mathrm{CO}}}{\left(f_C w_{[C]}\right)\cdot\left(\gamma_{(\mathrm{FeO})}w_{(\mathrm{FeO})}\frac{M_{\mathrm{slag}}}{M_{\mathrm{FeO}}}\right)}\tag{S68}$$

Based on Equations (S67) and (S68), the equilibrium concentrations $w^*_{[\mathrm{C}]}$ and $w^*_{(\mathrm{FeO})}$ at a temperature of 1873 K can be determined, as illustrated in **Equations (S69) and (S70)**.

$$w^*_{[\mathrm{C}]}=\frac{1}{10^{1.985}\cdot\gamma_{(\mathrm{FeO})}w^{\mathrm{b}}_{(\mathrm{FeO})}}\cdot\frac{M_{\mathrm{FeO}}}{M_{\mathrm{slag}}}\tag{S69}$$

$$w^*_{(\mathrm{FeO})}=\frac{1}{10^{1.985}\cdot f_{\mathrm{C}}w^{\mathrm{b}}_{[\mathrm{C}]}}\cdot\frac{M_{\mathrm{FeO}}}{M_{\mathrm{slag}}}\tag{S70}$$

By substituting Equations (S69) and (S70) into Equation (S66), an expression for the decarbonization rate, incorporating variables such as the carbon content of the droplets ($w^{\mathrm{b}}_{[\mathrm{C}]}$), the FeO content in the slag ($w^{\mathrm{b}}_{(\mathrm{FeO})}$), and the droplet diameter (*d*), is obtained, as represented in **Equation (S71)**.

$$\frac{\mathrm{d}w_{[\mathrm{C}]}}{\mathrm{d}t}=\begin{cases}\frac{1}{50}\cdot\sqrt{\frac{D_{\mathrm{FeO}}u_d}{\pi d^3}}\cdot\left(1-\frac{f_{\mathrm{solid}}}{f^*_{\mathrm{solid}}}\right)^{1.25}\cdot\left(1-f_{\mathrm{solid}}\right)^{2/3}\cdot\frac{\rho_{\mathrm{s}}}{\rho_{\mathrm{d}}}\cdot\left[w^{\mathrm{b}}_{(\mathrm{FeO})}-\frac{1}{96.6f_{\mathrm{C}}w^{\mathrm{b}}_{[\mathrm{C}]}}\cdot\frac{M_{\mathrm{FeO}}}{M_{\mathrm{slag}}}\right] & , \left(J_{\mathrm{FeO}}/J_C\right)\leqslant 1\\ \frac{3}{25}\cdot\sqrt{\frac{D_{\mathrm{C}}u_d}{\pi d^3}}\cdot\left(1-f_{\mathrm{solid}}\right)^{2/3}\cdot\left(w^{\mathrm{b}}_{[\mathrm{C}]}-\frac{1}{96.6\gamma_{(\mathrm{FeO})}w^{\mathrm{b}}_{(\mathrm{FeO})}}\cdot\frac{M_{\mathrm{FeO}}}{M_{\mathrm{slag}}}\right) & , \left(J_{\mathrm{FeO}}/J_C\right)>1\end{cases}\tag{S71}$$

Due to the dynamic nature of the kinetic parameters involved in the chemical reaction process, including the carbon content of the droplets ($w^{\mathrm{b}}_{[\mathrm{C}]}$), droplet diameter (*d*), and instantaneous droplet velocity (u_d), the finite difference method can be employed to calculate the variation in carbon content of a single metallic droplet during its flight through the emulsified slag under different operating conditions, as shown in **Figure S1**.

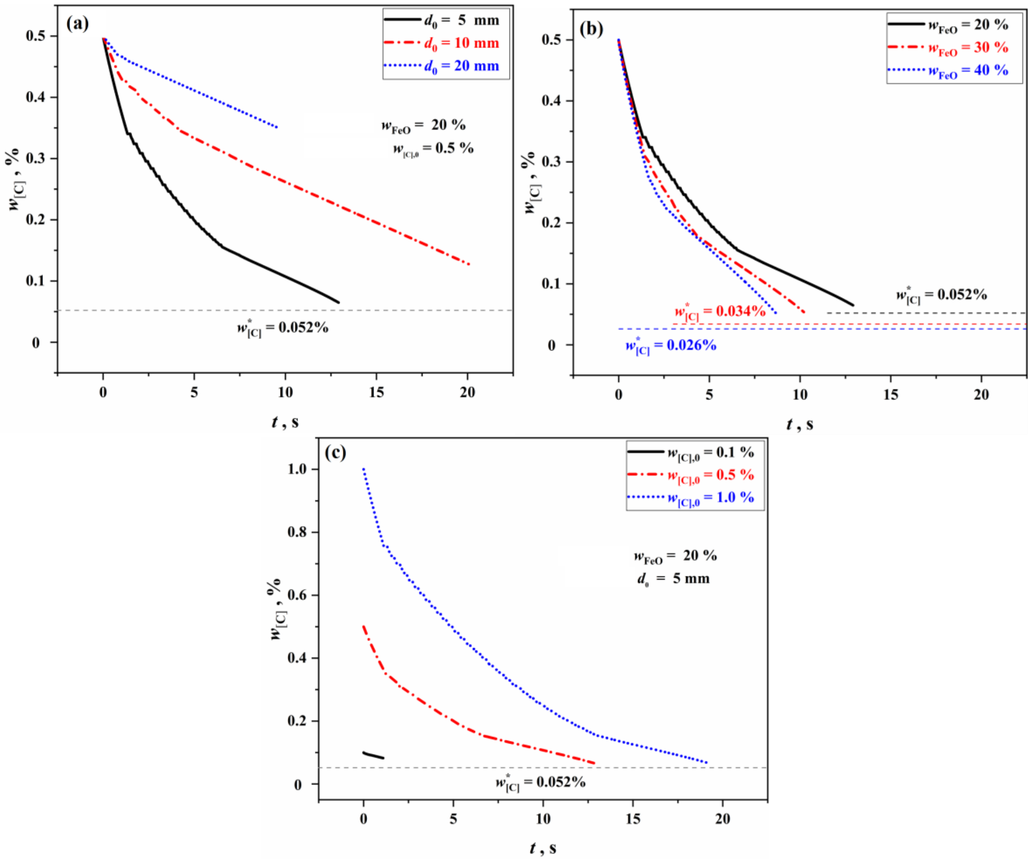

Figure S1. Change of carbon content for metal droplets with different (a) diameters of droplets; (b) FeO content of slag; (c) initial carbon content of droplets.

Figure S1 illustrates that small droplets with an initial diameter of $d_0 = 5\ \text{mm}$ exhibit a carbon content after reaction in the slag that is closer to the carbon content at the slag-metal equilibrium than that of larger droplets with an initial diameter of $d_0 \geq 10\ \text{mm}$. This indicates that, under equivalent conditions, small droplets undergo a greater degree of decarburization than large droplets; however, there is no direct correlation between the residence time of the droplets in the slag and their diameter. The results in Figure 8 indicate that droplets with identical initial diameters and carbon contents decarburize more rapidly in slag with high FeO content and have shorter residence times in the slag; however, the final carbon content at the end of the reaction is relatively similar, suggesting that the amount of decarburization in droplets is not significantly influenced by the FeO content in the slag. Figure 9 compares the decarburization amounts of metallic droplets with varying initial carbon contents. The results indicate that droplets with higher carbon content undergo more complete decarburization in the slag, while droplets with lower carbon content exhibit a slight reduction in their decarburization level. This phenomenon primarily arises because droplets with higher carbon content experience expansive suspension behavior in the slag due to a decarburization rate exceeding the critical value (r^*_C), resulting in a longer residence time and more thorough decarburization. During different stages of the converter blowing process, the particle size distribution of metal droplets generated by jet impact varies, and the initial carbon content of the droplets during the blowing process also differs. Consequently, the degree of decarburization of the droplets will vary, implying that the effective reaction quantity of the molten metal during the smelting process will also differ.

S3.2 Calculation of droplet generation and its particle size distribution

Li and Harris [9] developed a mathematical equation to describe the occurrence of droplet splashing, grounded in the Kelvin-Helmholtz instability theory. Droplet splashing occurs when the conditions specified in **Equation (S72)** are fulfilled.

$$\frac{\rho_g U_g^2}{2\sqrt{\rho_{\mathrm{m}} \sigma_{\mathrm{m}} g}} \geq 1 \tag{S72}$$

A relationship exists between the tangential velocity of the gas flow at the surface of the molten metal (U_g) and the center velocity of the free jet (U_j), as indicated in **Equation (S73)**.

$$U_g = \eta U_j \tag{S73}$$

According to the definitions of the blowing number provided by Subagyo and Brooks et al. [10], substituting Equation (S73) into Equation (S72) results in the transformation equation for $N_{\mathrm{B,T}}$, as illustrated in **Equation (S74)**.

$$N_{\mathrm{B,T}} = \eta^2 \cdot \frac{\rho_g U_j^2}{2} \cdot \frac{1}{\sqrt{\rho_l \sigma_l g}} \tag{S74}$$

Through the fitting of data from both cold (room temperature) and hot experiments, Subagyo and Brooks et al. [10] derived the equations for droplet generation, as presented in **Equations (S75) and (S76)**.

$$\frac{R_{\mathrm{B,T}}}{F_{\mathrm{G,T}}} = \frac{\left(N_{\mathrm{B,T}}\right)^{3.2}}{\left[2.6\times10^{6} + 2.0\times10^{-4}\left(N_{\mathrm{B,T}}\right)^{12}\right]^{0.2}} \tag{S75}$$

$$F_{\mathrm{G,T}} = F_{\mathrm{G}}^{\Theta} \cdot \frac{P^{\Theta}}{P} \cdot \frac{T}{T^{\Theta}} \tag{S76}$$

Related studies [11–13] have found that the particle size distribution of metal droplets generated by jet impingement in BOF follows the Rosin-Rammler-Sperling (RRS) function, as shown in **Equation (S77)**.

$$R = 100 \cdot \exp\left[-\left(\frac{d}{d'}\right)^{n}\right] \tag{S77}$$

S3.3 Relationship between effective decarburization rate of droplet and initial diameter

The effective decarburization rate (ΔC_{d}) of a single droplet is defined as presented in **Equation (S78)**.

$$\Delta C_{\mathrm{d}} = \frac{w_{[\mathrm{C}],0} - w_{[\mathrm{C}],\mathrm{end}}}{w_{[\mathrm{C}],0} - w_{[\mathrm{C}]}^{*}} \tag{S78}$$

Based on Equation (S78), the effective decarburization rates (ΔC_{d}) of droplets under relevant conditions are calculated, as shown in **Figures S2 to S4**. From Figures S2 to S4, it can be observed that the decarburization rate (ΔC_{d}) of droplets exhibits a significant correlation with the initial droplet diameter (d_0), FeO content in the slag (w_{FeO}), and the initial carbon content of the droplets ($w_{[\mathrm{C}],0}$). Specifically, in Figure S2, the effective decarburization rate of droplets shows a pronounced S-shaped decay trend as the diameter (d_0) increases; smaller droplet diameters correspond to higher effective decarburization rates, while droplets with diameters exceeding 60 mm exhibit effective decarburization

rates that are negligible. In Figure S3, when the FeO content in the slag is more than 10%, the effective decarburization rate of droplets is less affected by the FeO content in the slag. Smaller diameter droplets approach complete decarburization, but for larger diameter droplets, the effective decarburization rate slightly decreases with increasing FeO content. In Figure S4, when the initial carbon content of the droplets is more than 0.5%, the droplets are nearly completely decarburized and are not influenced by the carbon content. However, when the carbon content is less than 0.1%, the effective decarburization rate sharply decreases as the carbon content decreases.

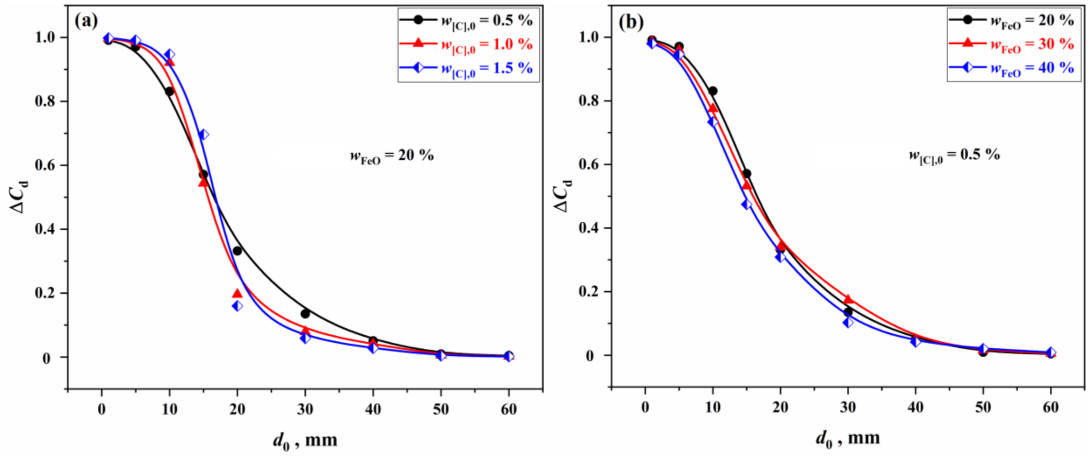

Figure S2. Decarburization rates of droplets with different initial diameter of droplets for different(a) initial carbon content of droplets; (b) FeO content of slag.

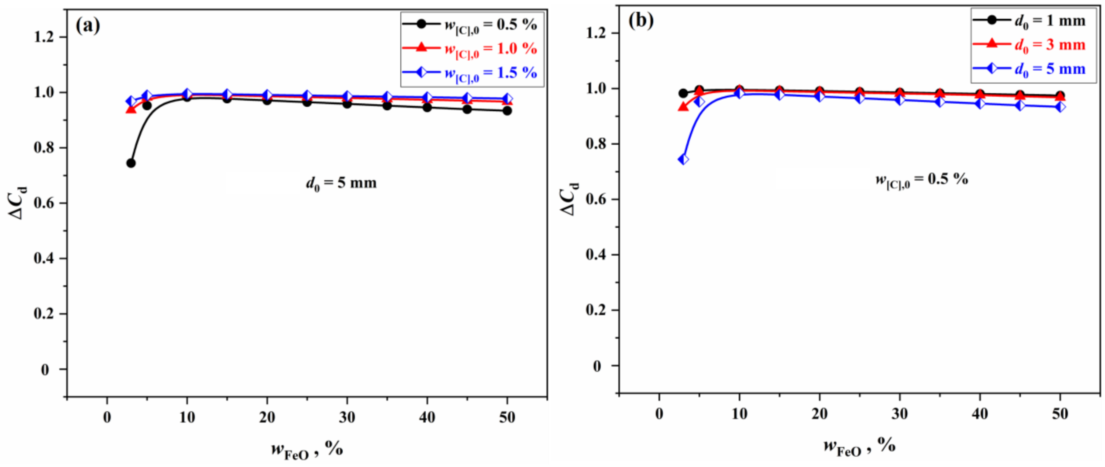

Figure S3. Decarburization rates of droplets with different FeO content of slag for different(a) initial carbon content of droplets; (b) initial diameter of droplets.

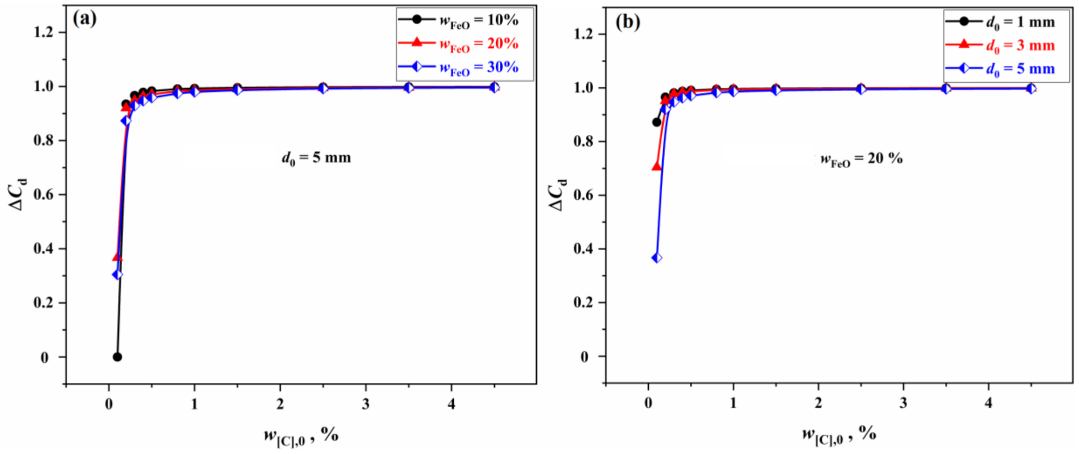

Figure S4. Decarburization rates of droplets with different initial carbon content of droplets for different(a) FeO content of slag; (b) initial diameter of droplets.

In this study, the effects of factors $w_{(\mathrm{FeO})}$ and $w_{[\mathrm{C}],0}$ are reflected in the thermodynamics of the reaction, while the influence of d0 must be considered as a kinetic factor in the calculation of droplet decarburization, obtained through regression fitting. Based on the results from Figures S4 to S6, the relationship between ΔC_{d} and d_0 is derived from Logistic function regression of the S-curve, as shown in **Figure S5**, leading to **Equation (S79)**.

$$\Delta C_{\mathrm{d}} = \frac{\Delta C_{\mathrm{d}}^{\max}}{1+\left(d_0/d_0^{0.5}\right)^{\psi}} \tag{S79}$$

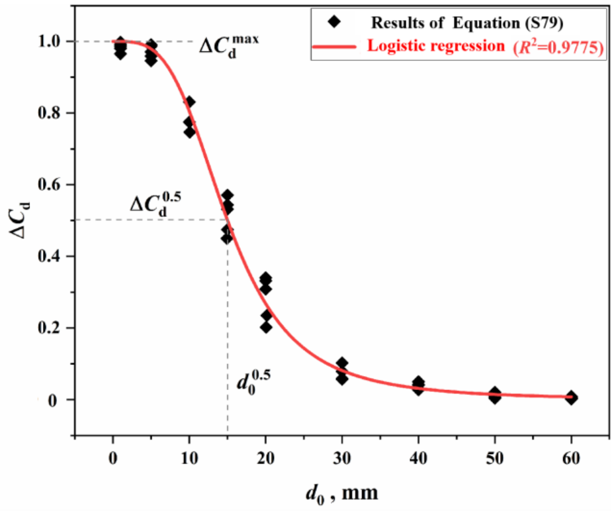

Figure S5. Relationship between effective decarburization rate and initial diameter of droplet.

S4 Oxygen ratio calculation for direct oxidation reaction and secondary combustion reaction of furnace gas in impact zone

The definition of the Post Combustion Ratio (PCR) in the BOF process is provided as shown in **Equation (S80)**.

$$PCR = \frac{\varphi_{\mathrm{CO_2}}}{\varphi_{\mathrm{CO}} + \varphi_{\mathrm{CO_2}}} = \frac{N_{\mathrm{CO_2}}}{N_{\mathrm{CO}} + N_{\mathrm{CO_2}}} = \frac{N_{\mathrm{CO_2}}}{N_{\mathrm{C}}} \tag{S80}$$

Based on the combustion reaction between CO and O_2, the ratio of the increase in CO_2 to the moles of O_2 consumed during secondary combustion is 2:1. Since the total moles of furnace gas remain constant before and after combustion (i.e., N_{C} remains unchanged), as shown in **Equation (S81)**.

$$PCR = \frac{N_{\mathrm{CO_2}}}{N_{\mathrm{C}}} = \frac{2N_{\mathrm{O_2}}^{\mathrm{GHZ}}}{2N_{\mathrm{O_2}}^{\mathrm{IZ}} \cdot x_{\mathrm{O}}^{\mathrm{C}}} = \frac{W_{\mathrm{O_2}}^{\mathrm{GHZ}}}{W_{\mathrm{O_2}}^{\mathrm{IZ}} \cdot x_{\mathrm{O}}^{\mathrm{C}}} \tag{S81}$$

By combining Equations (S80) and (S81), the proportion of oxygen consumed ($W_{\mathrm{O_2}}^{\mathrm{IZ}}$) in gas-liquid reactions within the impact zone and the oxygen used for secondary combustion ($W_{\mathrm{O_2}}^{\mathrm{GHZ}}$) to the total oxygen supply can be obtained, as shown in **Equations (S82) and (S83)**, respectively.

$$\frac{W_{\mathrm{O_2}}^{\mathrm{IZ}}}{W_{\mathrm{O_2}}^{\mathrm{tot}}} = \frac{1}{1 + PCR \cdot x_{\mathrm{O}}^{\mathrm{C}}} \tag{S82}$$

$$\frac{W_{\mathrm{O_2}}^{\mathrm{GHZ}}}{W_{\mathrm{O_2}}^{\mathrm{tot}}} = \frac{PCR \cdot x_{\mathrm{O}}^{\mathrm{C}}}{1 + PCR \cdot x_{\mathrm{O}}^{\mathrm{C}}} \tag{S83}$$

The total oxygen supply ($W_{\mathrm{O_2}}^{\mathrm{tot}}$) is known, specific values for $W_{\mathrm{O_2}}^{\mathrm{IZ}}$ and $W_{\mathrm{O_2}}^{\mathrm{GHZ}}$ can be obtained using the Equations (S82) and (S83).

For top-blown oxygen converters, the theoretical PCR can be calculated using **Equation (S84)**[14].

$$PCR = 0.1 \cdot \left(\frac{x - x_1}{d_{\mathrm{th}}} \right)^{0.3} - \left(\frac{x - x_1}{d_{\mathrm{th}}} \right)^{-0.7} + 0.01 \tag{S84}$$

In industrial production, it is essential to consider the impact of air entrainment at the furnace mouth on the original flue gas. The actual secondary combustion ratio of the original flue gas is determined by calculating the flue gas composition at the cold end of the flue duct, with specific calculation formulas provided in **Equations (S85) to (S87)**.

$$\varphi_{\mathrm{CO}}^{\mathrm{initial}} = \varphi_{\mathrm{CO}} + 2 \times \frac{21}{79} \times \left(100 - \varphi_{\mathrm{CO}} - \varphi_{\mathrm{CO_2}} \right) \tag{S85}$$

$$\varphi_{\mathrm{CO_2}}^{\mathrm{initial}} = \varphi_{\mathrm{CO_2}} - 2 \times \frac{21}{79} \times \left(100 - \varphi_{\mathrm{CO}} - \varphi_{\mathrm{CO_2}} \right) \tag{S86}$$

$$PCR^{\mathrm{initial}} = \frac{\varphi_{\mathrm{CO_2}}^{\mathrm{initial}}}{\varphi_{\mathrm{CO}}^{\mathrm{initial}} + \varphi_{\mathrm{CO_2}}^{\mathrm{initial}}} \tag{S87}$$

By substituting the actual or theoretical values of the secondary combustion ratio of the original flue gas into Equations (S82) and (S83), the proportions of oxygen used for the direct oxidation reactions in the impact zone and for the secondary combustion reactions in the flue gas can be obtained.

S5 Melting reaction of scrap in metal mixing zone

There are two main melting mechanisms of scrap steel in molten bath: carbon concentration-driven melting and heat-driven melting [6]. The specific descriptions are as follows.

(1) Carbon concentration-driven melting: If the temperature of the molten metal pool is lower than the melting temperature of the scrap steel, the melting process of the scrap is governed by the difference in carbon concentration between the molten pool and the scrap. Carbon diffuses from the high-carbon molten pool to the low-carbon scrap steel, causing the carbon content on the surface of the scrap to increase. This results in a reduction in its melting temperature, leading to its melting. The model for carbon concentration-driven melting of scrap steel is shown in **Equation (S88)**.

$$-\frac{\mathrm{d}r}{\mathrm{d}t} = k_{\mathrm{m}} \cdot \ln\left(1 + \frac{C_{\mathrm{HM}} - C_{\mathrm{liq}}}{C_{\mathrm{liq}} - C_{\mathrm{scrap}}} \right) \tag{S88}$$

(2) Heat-driven melting: If the temperature of the molten bath is higher than the melting temperature of the scrap steel, the melting process of the scrap is determined by the temperature difference between the molten metal and the scrap. The heat-driven melting model of scrap steel is shown in **Equation (S89)**.

$$-\frac{\mathrm{d}r}{\mathrm{d}t}=\frac{h_{\mathrm{m}}}{\rho_{\mathrm{scrap}}}\cdot\frac{\left(T_{\mathrm{HM}}-T_{\mathrm{liq}}\right)}{L_f+\left(T_{\mathrm{HM}}-T_{\mathrm{liq}}\right)\cdot c_p} \tag{S89}$$

The heat transfer coefficient (h_{m}) of the molten bath is calculated using the formula presented in **Equation (S90)**.

$$h_{\mathrm{m}}=5000\cdot\left(\varepsilon_{\mathrm{tot}}\right)^{0.3} \tag{S90}$$

S6 Dissolution reaction of flux in the mixed slag zone

The dissolution rates of CaO and MgO fluxes in slag are calculated according to **Equation (S91)** and **Equation (S92)**, respectively [15,16].

$$-\frac{\mathrm{d}r_{\mathrm{CaO}}}{\mathrm{d}t}=k_{\mathrm{s}}\cdot\frac{\rho_{\mathrm{s}}}{\rho_{\mathrm{CaO}}}\cdot\frac{\left(\%\mathrm{CaO}_{\mathrm{sat}}-\%\mathrm{CaO}_{\mathrm{slag}}\right)}{100}=k_{\mathrm{s}}\cdot\frac{\rho_{\mathrm{s}}}{\rho_{\mathrm{CaO}}}\cdot\frac{\left(\Delta\%\mathrm{CaO}\right)}{100} \tag{S91}$$

$$-\frac{\mathrm{d}r_{\mathrm{MgO}}}{\mathrm{d}t}=k_{\mathrm{s}}\cdot\frac{\rho_{\mathrm{s}}}{\rho_{\mathrm{MgO}}}\cdot\frac{\left(\%\mathrm{MgO}_{\mathrm{sat}}-\%\mathrm{MgO}_{\mathrm{slag}}\right)}{100}=k_{\mathrm{s}}\cdot\frac{\rho_{\mathrm{s}}}{\rho_{\mathrm{MgO}}}\cdot\frac{\left(\Delta\%\mathrm{MgO}\right)}{100} \tag{S92}$$

From Equations (S91) and (S92), it can be seen that the dissolution rate of flux in slag is determined by the saturation concentration difference and the effective mass transfer coefficient in the slag.

Symbol description

ΔG_i : Gibbs free energy of unit moles of O_2 reacting with element i in the molten metal, J/mol, $i=$ C, Si, Mn, Fe;

$x_{\mathrm{O_2}}^{i}$: The proportion of oxygen in unit moles of O_2 that reacts with element i;

W_j : Mass of component *j* in flue gas or slag ($j=$ CO, CO_2, SiO_2, MnO, FeO), kg;

M_j : Molar mass of component j in flue gas or slag, kg/mol;

$W_{\mathrm{metal}}^{\mathrm{IZ}}$: The quality of molten metal involved in the reaction in the impact zone, kg;

R_i : Mass transfer rate of molten metal side component *i* at reaction interface, mol/s, $i=$[Fe], [C], [Si], [Mn], [P], [O];

R_j : Mass transfer rate of molten slag side component *j* at reaction interface, mol/s, $j=\left(\mathrm{SiO_2}\right),\left(\mathrm{MnO}\right),\left(\mathrm{P_2O_5}\right),\left(\mathrm{FeO}\right)$;

A_{sm} : The area of the slag-metal reaction interface, m^2;

k_i : Mass transfer coefficients of molten metal side component *i* at reaction interface, m/s;

k_j : Mass transfer coefficients of molten slag side component *j* at reaction interface, m/s;

ρ_{m} : Density of molten metal, kg/m^3;

ρ_{m} : Density of slag, kg/m^3;

C_i^{b} : Molar concentration of component *i* in molten metal, mol/m^3;

C_i^{*} : Molar concentration of component *i* at reaction interface, mol/m^3;

C_j^{b} : Molar concentration of component *j* in slag, mol/m^3;

C_j^{*} : Molar concentration of component *j* at reaction interface, mol/m^3;

w_i^{b} : Mass concentration of component *i* in molten metal, %;

w_i^*: Mass concentration of component *i* at reaction interface, %;

w_j^b: Mass concentration of component *j* in slag, %;

w_j^*: Mass concentration of component *j* at reaction interface, %;

M_i: Molar mass of components *i*, kg/mol;

M_j: Molar mass of components *j*, kg/mol;

$a_{[i]}$: Activity of component *i* in molten metal;

$a_{(j)}$: Activity of component *j* in slag;

$f_{[i]}$: Activity coefficient of component *i* in molten metal;

$\gamma_{(j)}$: Activity coefficient of component *j* in slag;

$w_{[i]}^*$: Mass concentration of component *i* in molten metal at the reaction interface, %;

$X_{(j)}^*$: Molar fraction of component j in slag at the reaction interface, %;

$P_{\mathrm{CO}}/P^{\Theta}$: CO partial pressure in furnace gas;

f_{C}: Activity of carbon in molten metal;

i: Dissolved elements in molten metal, $i = \mathrm{C, Si, Mn, P, O}$;

$w_{[i]}$: Mass concentration of element *i* in molten metal, %;

e_{C}^i: Interaction coefficient of element *i* on carbon;

a_j: Activity of component *j* in slag;

γ_j: Activity coefficient of component *j* in slag;

X_j: Molar ratio of component *j* in slag;

B: Basicity of slag, $B = w_{(\mathrm{CaO})}/w_{(\mathrm{SiO_2})}$;

u_l: Circulation renewal rate of molten metal in molten bath under single top blowing condition, m/s;

u_{bottom}: Circulation renewal rate of molten metal under single bottom blowing condition, m/s;

θ: Angle of the pit shape, $\theta = \tan^{-1}\left(\frac{r_{\mathrm{cav}}}{h_{\mathrm{cav}}}\right)$;

h_{cav}: Depth of the pit, m;

Q_{B}: Bottom blow flow, m^3/s;

z_{m}: Vertical height from the bottom air outlet, m;

Q_{B}^*: Dimensionless bottom blow flow;

z_{m}^*: Vertical height from bottom blow outlet;

H_{bath}: Depth of molten bath, m;

g: Gravitational acceleration, m^2/s;

$\varepsilon_{\mathrm{tot}}$: The comprehensive stirring energy of top and bottom blowing, W/m^3;

ε_{T}: Top blowing stirring energy, W/m^3;

ε_{B}: Bottom blowing stirring energy, W/m^3;

Q_{T}: Gas flow rate of top blowing, Nm^3/min;

Q_B : Gas flow rate of bottom blowing, Nm^3/min;

T_m : The temperature of molten metal, K;

T_g : The temperature of the bottom blowing gas, K;

V_m : Volume of molten metal in molten bath, m^3;

ρ_m : Density of molten metal in molten bath, kg/m^3;

H_{bath} : Depth of molten metal in molten bath, m;

η : Mixing efficiency;

α : Oxygen gun orifice angle, $^{\circ}$;

M_{O_2} : Molar mass of top blown oxygen, kg/mol;

N : Number of oxygen lance nozzle, $N = 4$ in this study;

d_e : Diameter of the oxygen lance nozzle outlet, m;

x : Position of oxygen lance, m;

λ : Stirring efficiency of top blowing jet energy, usually λ is 0.1;

P_{CO} : Partial pressure of CO gas, P_{CO} is approximately 1;

a_C : Activity of C in molten metal;

$a_{(FeO)}$: Activity of FeO in slag;

f_C : Activity coefficient of C in molten metal;

$\gamma_{(FeO)}$: Activity coefficient of FeO in slag;

$w_{[C]}$: Mass fraction of C in molten metal, %;

$w_{(FeO)}$: Mass fraction of FeO in slag, %;

M_{slag}/M_{FeO} : Ratio of total molar mass of slag to molar mass of FeO, M_{slag}/M_{FeO} is between 0.8 and 0.9 in this study.

ρ_g : Density of gas, kg/m^3;

ρ_m : Density of molten metal, kg/m^3;

σ_m : Surface tension of molten metal, N/m;

U_g : Tangential velocity of air flow on molten metal surface, m/s;

U_j : Free jet velocity at impact point, m/s;

η : A dimensionless constant relating to the physical properties of liquid, the η of molten metal in converter steelmaking is 0.4472[13];

ρ_l : Density of liquid phase, kg/m^3;

σ_l : Surface tension in the liquid phase, N/m;

$R_{B,T}$: Amount of droplet generation, kg/s;

$F_{G,T}$: Gas flow rate of top blowing, m^3/s;

$N_{B,T}$: Blowing number;

F_G^{Θ} : Gas flow rate of top blowing under standard condition, Nm^3/s;

P^{Θ} : Air pressure under standard condition, Pa;

T^{Θ} : Temperature under standard condition, K.

R: Weight percentage of droplets with a diameter greater than *d* in the total droplet population, %;

d: Diameter of droplet, mm;
d': Distribution characteristic diameter of droplet, mm;
n: Parameter that characterizes the uniformity of droplet size distribution, this is related to material properties;
$w_{[C],0}$: Initial carbon content of the droplet as it flies out of the molten bath, %;
$w_{[C],end}$: Carbon content of droplet falling from emulsified slag phase to molten bath, %;
$w_{[C]}^{*}$: Carbon content when the droplet and slag completely react to reach equilibrium, %;
ΔC_{d}^{max}: The maximum effective decarburization rate of the droplet, ΔC_{d}^{max} is 1 when completely decarbonized;
d_0: Initial diameter of the droplet, mm;
$d_0^{0.5}$: Droplet diameter when the effective decarburization rate is half of the maximum, the fitting result of this study is $d_0^{0.5} = 15$ mm.
ψ: Attenuation coefficient, the fitting result of this study is $\psi = 3.5$;
φ_{CO}: Volume percentage of CO in furnace gas, %;
φ_{CO_2}: Volume percentage of CO_2 in furnace gas, %;
N_{CO}: Mole number of CO in furnace gas, mol;
N_{CO_2}: Mole number of CO_2 in furnace gas, mol;
N_{C}: Total mole number of C in furnace gas, mol;
$N_{O_2}^{GHZ}$: Mole number of oxygen for secondary combustion of furnace gas, mol;
$N_{O_2}^{IZ}$: Mole number of oxygen used in the impact zone reaction, mol;
x_{O}^{C}: Proportion of oxygen used for decarburization in the gas-liquid reaction in the impact zone;
x_1: Length of jet core segment, m;
d_{th}: Throat diameter of oxygen lance nozzle, m;
$PCR^{initial}$: Secondary combustion rate of the original furnace gas, that is, the proportion of CO2 in the original furnace gas;
$\varphi_{CO}^{initial}$: Percentage content of CO in the original furnace gas, %;
$\varphi_{CO_2}^{initial}$: Percentage content of CO_2 in the original furnace gas, %;
φ_{CO}: Percentage content of CO in flue gas at cold end of flue, %;
φ_{CO_2}: Percentage content of CO_2 in flue gas at cold end of flue, %;
r: Radius of scrap steel, m;
k_m: Mass transfer coefficient of molten metal, m/s;
C_{liq}: Carbon mass concentration when the liquidus temperature is the surface temperature of scrap steel, %;
C_{HM}: Carbon mass concentration of molten metal, %;
C_{scrap}: Carbon mass concentration of scrap steel, %;
h_m: Heat transfer coefficient of molten metal, $W(m^{-2} \cdot K^{-1})$;
L_f: Latent heat of fusion of scrap steel, $J \cdot kg^{-1}$;
c_p: Specific heat capacity, $J(kg^{-1} \cdot K^{-1})$;
ρ_{scrap}: Density of scrap steel, $kg \cdot m^{-3}$;
T_{HM}: Temperature of the molten bath, K;

T_{liq} : Liquidus temperature of scrap steel surface, K;

k_{s} : Effective mass transfer coefficient in slag, m/s;

$-\frac{\mathrm{d}r_{\mathrm{CaO}}}{\mathrm{d}t}$: Dissolution rate of CaO flux, m/s;

$-\frac{\mathrm{d}r_{\mathrm{MgO}}}{\mathrm{d}t}$: Dissolution rate of MgO flux, m/s;

$\%\mathrm{CaO}_{\mathrm{sat}}$: Saturation concentration of CaO in slag, %;

$\%\mathrm{MgO}_{\mathrm{sat}}$: Saturation concentration of MgO in slag, %;

$\%\mathrm{CaO}_{\mathrm{slag}}$: Actual concentration of CaO in slag, %;

$\%\mathrm{MgO}_{\mathrm{slag}}$: Actual concentration of MgO in slag, %;

$\Delta\%\mathrm{CaO}$: Concentration difference between saturation concentration and actual concentration of CaO in slag, %;

$\Delta\%\mathrm{MgO}$: Concentration difference between saturation concentration and actual concentration of MgO in slag, %;

ρ_{CaO} : Density of CaO flux, kg/m^3;

ρ_{MgO} : Density of MgO flux, kg/m^3.

References

1. Huang, X.H. *Principle of Ferrous Metallurgy*, 4th ed.; Metallurgical Industry Press: Beijing, China, 2013.
2. Hino, M.; Ito, K. *Thermodynamic data for steelmaking*, Tohoku University Press: Sendai, Japan, 2010.
3. Hwang, H. Y.; Irons, G. A. A water model study of impinging gas jets on liquid surfaces. *Metall. Mater. Trans. B* **2012**, 43, 302–315.
4. Krishnapisharody, K.; Irons, G. A. An analysis of recirculatory flow in gas-stirred ladles. *Steel Res. Int.* **2010**, 81, 880–885.
5. Van Ende, M.A.; Jung, I.H. A kinetic process simulation model for basic oxygen furnace (BOF): Importance of slag chemistry for BOF operation. *CAMP-ISIJ* **2015**, *28*, 527–530.
6. Lytvynyuk, Y.; Schenk, J.; Hiebler, M.; Sormann, A. Thermodynamic and kinetic model of the converter steelmaking process. Part 1: The description of the BOF model. *Steel Res. Int.* **2014**, *85*, 537–543.
7. Kitamura, S.Y.; Kitamura, T.; Shibata, K.; Mizukami, Y.; Mukawa, S.; Nakagawa, J. Effect of stirring energy, temperature and flux composition on hot metal dephosphorization kinetics. *ISIJ Int.* **1991**, *31*, 1322–1328.
8. Lin, W.H. Study on Process Behavior Analysis and Decarburization Control of BOF Steelmaking. Ph.D. Thesis, University of Science and Technology Beijing, Beijing, China, 2023.
9. Li, R.; Harris, R. L. Interaction of gas jets with model process liquids. In Proceedings of the Pyrometallurgy 95, London, UK, 31 May 1995; pp. 107-124.
10. Subagyo; Brooks, G. A.; Coley, K. S.; Irons, G.A. Generation of droplets in slag-metal emulsions through top gas blowing. *ISIJ Int.* **2003**, 43, 983–989.
11. Cicutti, C.; Valdez, M.; Pérez, T.; Petroni, J.; Gomez, A.; Donayo, R.; Ferro, L. Study of slag-metal reactions in an LD-LBE converter. In Proceedings of the 6th International Conference on Molten Slags, Fluxes and Salts, Helsinki, Finland, 12–17 June 2000; pp. 367–384.
12. Koria, S.C.; Lange, K.W. A new approach to investigate the drop size distribution in basic oxygen steelmaking. *Metall. Mater. Trans. B* **1984**, 15, 109–116.
13. Deo, B.; Boom, R. *Fundamentals of Steelmaking Metallurgy*; Prentice Hall International: London, UK, 1993.
14. Hirai, M.; Tsujino, R.; Mukai, T.; Harada, T.; Omori, M. The Mechanism of post combustion in converter. *Tetsu–Hagane* **1987**, *73*, 1117–1124.
15. Kitamura, S.Y.; Shibata, H.; Maruoka, N. Kinetic model of hot metal dephosphorization by liquid and solid coexisting slags. *Steel Res. Int.* **2008**, 79, 586–590.
16. Pahlevani, F.; Kitamura, S.; Shibata, H.; Maruoka, N. Simulation of steel refining process in converter. *Steel Res. Int.* **2010**, 81, 617–622.
